# Cell-Intrinsic Control of Interneuron Migration Drives Cortical Morphogenesis

**DOI:** 10.1016/j.cell.2018.01.031

**Published:** 2018-02-22

**Authors:** Carla G. Silva, Elise Peyre, Mohit H. Adhikari, Sylvia Tielens, Sebastian Tanco, Petra Van Damme, Lorenza Magno, Nathalie Krusy, Gulistan Agirman, Maria M. Magiera, Nicoletta Kessaris, Brigitte Malgrange, Annie Andrieux, Carsten Janke, Laurent Nguyen

**Affiliations:** 1 GIGA-Neurosciences, University of Liège, C.H.U. Sart Tilman, Liège 4000, Belgium; 2 Center for Brain and Cognition, Department of Information and Technology, Universitat Pompeu Fabra, Calle Ramon Trias Fargas 25-27, Barcelona 08005, Spain; 3 VIB-UGent Center for Medical Biotechnologie, VIB, 9000 Ghent, Belgium; 4 Department of Biochemistry, Ghent University, 9000 Ghent, Belgium; 5 Wolfson Institute for Biomedical Research and Department of Cell and Developmental Biology, University College London, London, UK; 6 Institut Curie, CNRS UMR3348, PSL Research University, Centre Universitaire, 91400 Orsay, France; 7 Université Grenoble Alpes, Grenoble Institut des Neurosciences, GIN, Inserm, U1216, 38000 Grenoble, France

**Keywords:** neurogenesis, glutamylation, carboxypeptidase, corticogenesis, actomyosin, cytoskeleton, MLCK

## Abstract

Interneurons navigate along multiple tangential paths to settle into appropriate cortical layers. They undergo a saltatory migration paced by intermittent nuclear jumps whose regulation relies on interplay between extracellular cues and genetic-encoded information. It remains unclear how cycles of pause and movement are coordinated at the molecular level. Post-translational modification of proteins contributes to cell migration regulation. The present study uncovers that carboxypeptidase 1, which promotes post-translational protein deglutamylation, controls the pausing of migrating cortical interneurons. Moreover, we demonstrate that pausing during migration attenuates movement simultaneity at the population level, thereby controlling the flow of interneurons invading the cortex. Interfering with the regulation of pausing not only affects the size of the cortical interneuron cohort but also impairs the generation of age-matched projection neurons of the upper layers.

## Highlights

• CCP1 controls MLCK activity by processing its polyglutamate carboxy-terminal
• CCP1 controls the stereotypic two-stroke cycle of cortical interneuron migration
• Asynchronous pausing during interneuron migration controls cortical invasion
• Interneuron cortical invasion modulates generation of age-matched projection neurons

## Graphical Abstract

**Figure:**
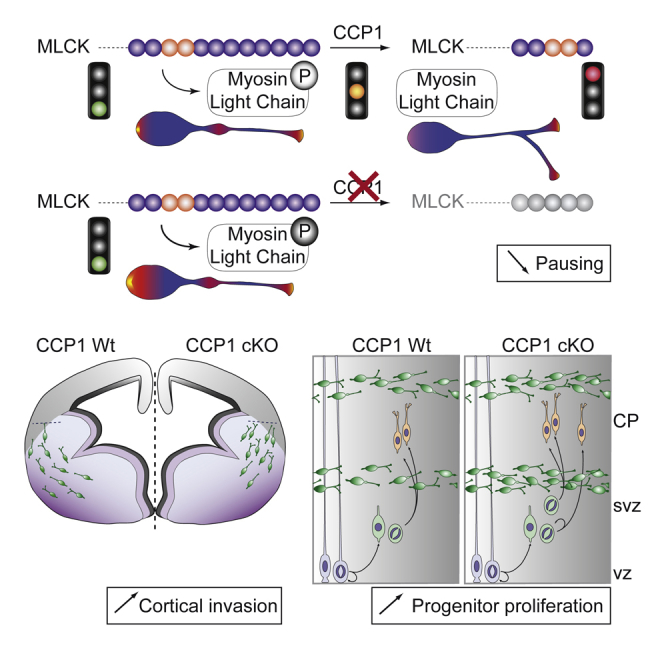

## Teaser

The “stop and go” movement of individual migrating cortical interneurons is critical for ensuring a proper rate of cortical invasion for the entire population.

## Introduction

Cell migration has a fundamental role in the formation of complex tissue structures, such as the brain, which involves a precise temporal and spatial migration of different cell types. Cellular models have demonstrated that the nature of the cell movement can influence population dynamics (Barber et al., 2015, Villar-Cerviño et al., 2013). Migration brings together projection neurons (PNs) and interneurons (INs) in the cerebral cortex: PNs migrate radially within the cortical wall, whereas INs originate from the ventral forebrain and reach the cortex by moving along tangential paths. In contrast to a steadier motion of PNs undergoing somal translocation or locomotion on radial glial fibers to settle in the cortical plate, INs undergo a stereotypic two-stroke cycle of migration, switching from sharp periods of movement to pauses (Bellion et al., 2005). At the molecular level, the migration of cINs and PNs relies on the microtubule (MT) and actomyosin cytoskeletons that integrate extracellular signals governing directed migration and that generate the forces required for cell movement. MT polymerization is required for the extension of the leading process whereas actomyosin dynamic directs the centrosomal displacement and nuclear movement referred to as nucleokinesis (Bellion et al., 2005). It is intriguing how these two neuronal populations, despite using similar cellular machineries, migrate using distinct types of choreography. Moreover, it remains unclear how these different migratory behaviors contribute to the exquisite organization of the cerebral cortex. Several studies have explored the contribution of cytoskeleton-regulatory proteins to tangential migration (Godin et al., 2012, Kappeler et al., 2006), but none of them provided a physiological significance to the saltatory motion of cIN migration.

Post-translational modifications (PTM) of proteins are important for central cellular processes such as cell migration. Indeed, genetic manipulations preventing the activity of PTM enzymes have been associated with neuronal migration defects (Creppe et al., 2009, Hahn et al., 2013), suggesting that some key proteins involved in cell motility undergo post-translational regulation. Here, we explored the contribution of protein polyglutamylation, which occurs on a variety of proteins (Tanco et al., 2013, van Dijk et al., 2008). We found that several enzymes regulating (de)glutamylation from the tubulin tyrosine ligase-like family (TTLLs) or cytosolic carboxypeptidases (CCPs) (Janke et al., 2005, van Dijk et al., 2007) are expressed in the subpallium where newborn cINs accumulate before entering into the cortex. One striking feature of CCPs is that they remove not only glutamate side chains generated by polyglutamylation but also gene-encoded polyglutamate stretches at carboxy termini of proteins (Rogowski et al., 2010, Tort et al., 2014). Here, we show that the genetic disruption of *Ccp1* (*Agtpbp1*), the major deglutamylating enzyme expressed in cINs, modifies their migration mode without affecting average velocity. At the molecular level, we found that loss of CCP1 induces cytoskeletal changes leading to a significant decrease of pause duration, thereby converting the saltatory migration of cINs into a treadmill-like motion. This switch of migration mode reduces randomness in movement timings at the population level, thereby leading to higher recruitment of cINs into the cortical wall. We also show that the cortex detects the overflow of cINs and fine-tunes the size of the age-matched PN population by scaling cortical progenitor proliferation. Altogether, our study demonstrates that a tight intrinsic regulation of pausing in migrating cINs has a pivotal role in controlling neocortex histogenesis.

## Results

### Conditional Loss of Ccp1 in Cortical Interneurons Converts Saltatory Migration into a Steadier Motion

**Figure 1:**
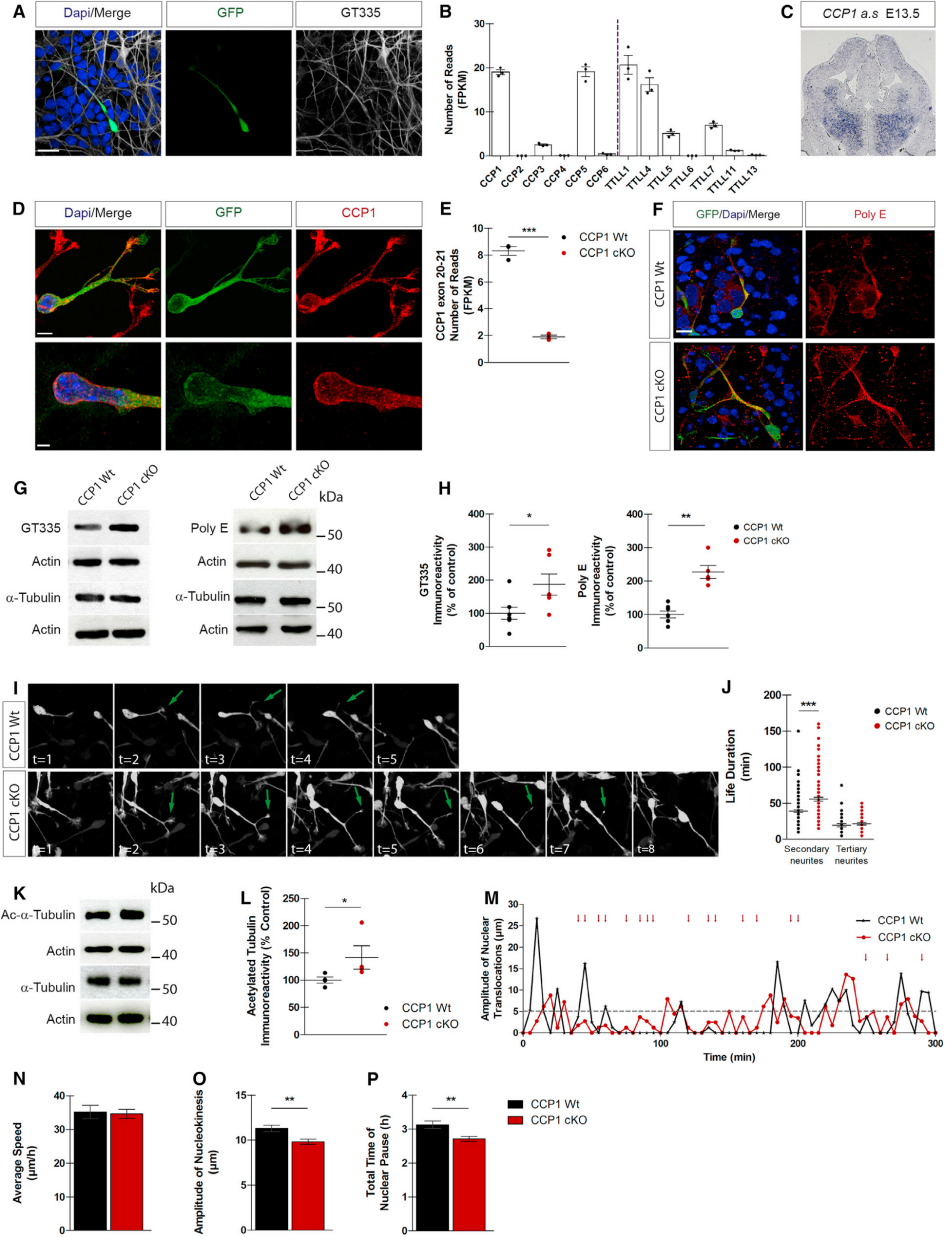
CCP1 Promotes the Saltatory Migration of cINs (A) Immunodetection of polyglutamate side chains (GT335 antibody) on cINs explants. cINs express Cre-GFP and nuclei are counterstained with DAPI. The white arrow points the leading process. Scale bar, 10 μm. (B) Normalized expression levels of *CCP* and *TTLL* mRNAs in E13.5 WT cINs, n = 3 embryos *per* group from 3 females. (C) ISH of *Ccp1* on a coronal section of E13.5. (D) Subcellular distribution of CCP1 (red) in WT migrating cINs. Scale bars, 5 μm (top), 2 μm (bottom). (E) Normalized expression levels of *Ccp1* exons 20 and 21 in FACS-purified cINs from 3 E13.5 CCP1 WT or cKO mouse embryos. (F) Glutamylation levels (PolyE or GT335 antibodies) on tubulin extracted from GEs of CCP1 WT and cKO embryos at E13.5. (G) PolyE and GT335 immunoreactivity normalized on tubulin and actin and expressed as percentage of control, n = 5–7 embryos per group, non-parametric t test, p < 0.05. (H) Immunodetection of polyglutamate stretches (PolyE antibody) on cIN explants. INs from CCP1 WT and cKO express Cre-GFP and Dapi^+^ nuclei. Scale bar, 10 μm. (I) Representative time series of time-lapse acquisition of migrating cINs from CCP1 WT and cKO embryos. cINs express GFP and secondary neurites are pointed by green arrows. (J) Neurites life duration, n = 26–127 cells per group from at least 3 independent cultures, parametric t test, p < 0.001. (K) Tubulin acetylation levels on protein extracts from E13.5. CCP1 WT and cKO GEs. (L) Normalized acetylation immunoreactivity normalized, n = 4 embryos per group, p < 0.05. (M) Nuclear displacement of representative migrating cINs from CCP1 WT and cKO E13.5 embryos. The traveled distance between two time points is plotted and every displacement above 5 μm (gray dotted line) is a nucleokinesis. Red arrows indicate sliding movement in CCP1 cKO. A nuclear pause corresponds to no displacement between two time points. (N–P) Average speed (N), amplitude (O) of nucleokinesis, and time of nuclear pause (P) of CCP1 WT and cKO cINs measured in E13.5 organotypic slice culture (see also Figure S1; n = 45–78 cells in at least 3 embryos from at least 3 females; p < 0.01). All graphs contain bars representing the SEM.

**Figure S1:**
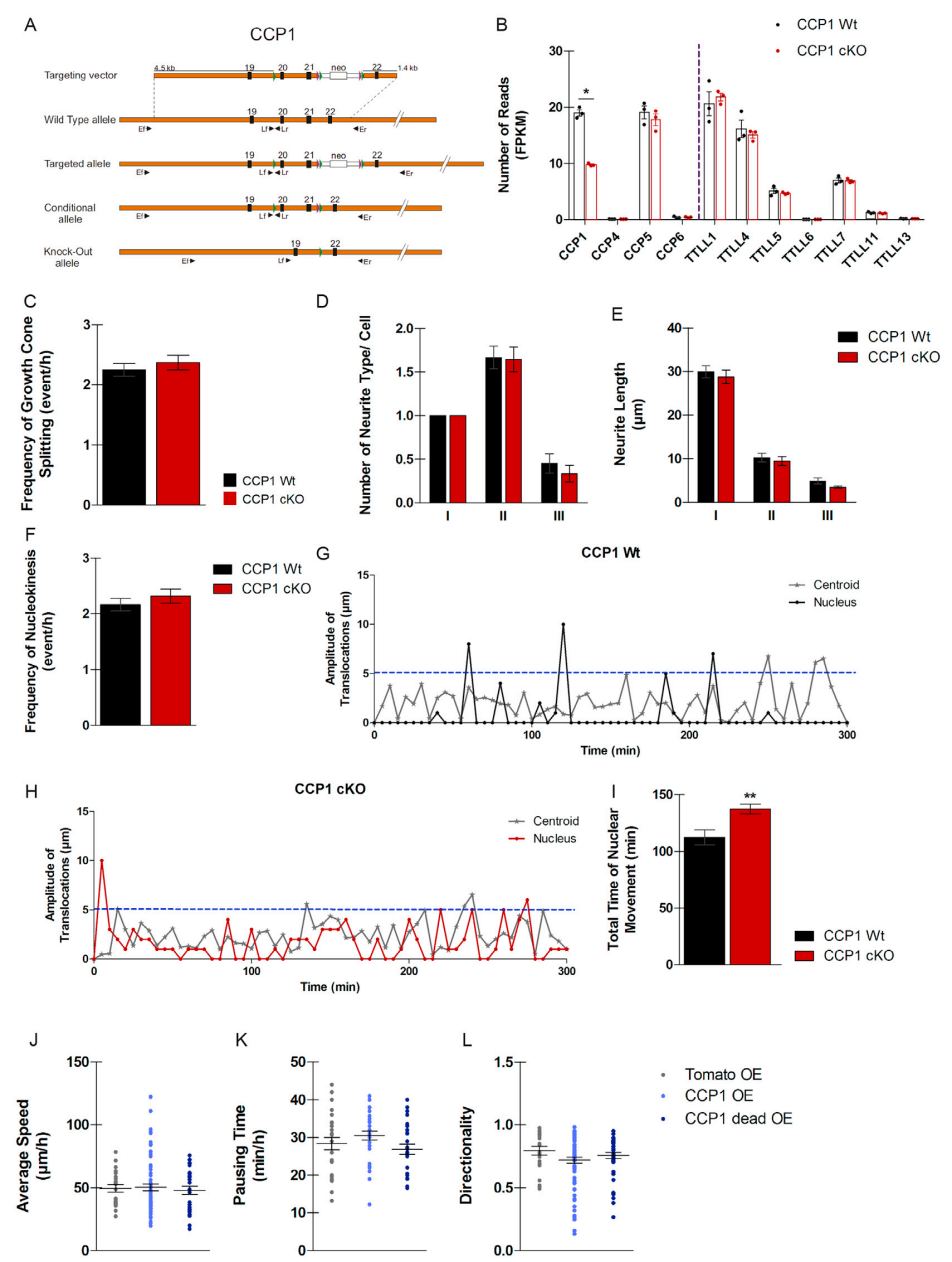
Assessment of Migration Parameters in Cortical Interneurons Lacking *Ccp1* Expression, Related to Figure 1 (A) Schematic representation of the targeting vector used and all possible alleles for *Ccp1* gene. Orange bar: genomic DNA. Black boxes: exons with their corresponding number. Green and purple arrowheads: LoxP and Flp sequences, respectively. White bar: neo cassette, with the neomycin resistance gene (white box). Blue lines: zone of sequence homology for homologous recombination, with the corresponding size in kbp. Black arrowheads: primers used for the PCR genotyping. (B) Normalized expression levels of *CCP* and *TTLL* mRNAs from extracted RNA of FACS E13.5 CCP1 WT and CCP1 cKO GEs. Values are expressed as FPKM, n = 3 embryos from independent female donors. (C–F) Time-lapse experiments of CCP1 WT and CCP1 cKO cINs from explant co-cultures. Quantification of growth cone splitting (emergence of a secondary branch from the growth cone) (C), average number of different neurite types on cINs during migration (I = primary neurites, II = secondary and III = tertiary neurites) (D), neuritic length (E) and frequency of nucleokinesis (F), respectively, n = 26-52 cells from at least 3 independent cultures, two-way ANOVA. (G and H) Representative examples of CCP1 WT and CCP1 cKO nucleus (G) and centroid displacements (H) measured by time-lapse acquisition in E13.5 GEs explants. The traveled distance between two time points is plotted and every displacement above 5μm (blue dotted line) is considered as a nucleokinesis. (I) Quantification of the total time of nuclear movement in CCP1 WT and CCP1 cKO measured in time-lapse acquisition of E13.5 organotypic slice culture. (J–L) Time-lapse analysis of cINs in explant co-culture showing average speed (J), pausing time (K), and directionality (L) after overexpression of *Ccp1* (CCP1 OE), its catalytic dead form (CCP1dead OE), or a control (Tomato OE), n = 22-78 cells from at least 3 independent cultures, one-way ANOVA. All graphs contain bars representing SEM.

Glutamylation is a major PTM of microtubules (MTs) in neurons that serves as a signaling code for modulating protein function according to the size of the polyglutamate chains (Janke, 2014). The length and complexity of glutamate side chains are controlled by the coordinated activity of enzymes performing glutamylation (TTLL proteins) or deglutamylation (CCP proteins). TTLL4, TTLL5, and TTLL7 add the branching point glutamate, while TTLL1, TTLL6, TTLL11, and TTLL13 promote glutamate chain elongation (Janke et al., 2005, van Dijk et al., 2007). Similarly, CCP5 has higher specificity in removing the branching point glutamate on lateral chains while CCP1, CCP2–CCP4, and CCP6 deglutamylate lateral or gene-encoded polyglutamate chains (Rogowski et al., 2010, Tort et al., 2014). Here, we found that the leading process of migrating cINs showed detectable levels of glutamylation (Figure 1A). *Dlx5/6*-positive subpallial structures express several *Ttll* and *Ccp* genes (Figure 1B). *Ccp1*, which codes for the main glutamate chain deglutamylase, is specifically enriched in the SVZ and mantle zone of GEs where cINs migrate (Figure 1C). CCP1 is mainly detected in the growth cones and around the nucleus (Figure 1D). According to its expression pattern, and because targeting CCP5 or TTLL1 have a major impact on MTs function by dramatically modifying glutamylation patterns, we conditionally invalidated *Ccp1* from newborn cINs. *Ccp1*^lox/lox^ mice were crossed with *Dlx5-6* Cre-GFP mice (Stenman et al., 2003) to remove the catalytic domain of CCP1 in most forebrain GABAergic neurons (further named CCP1 conditional knockout [cKO]; Figures 1E and S1A). *Ccp1* invalidation was not genetically compensated by changes in expression level of other *Ttlls* and *Ccps* (Figure S1B). Western blotting showed accumulation of hyperglutamylated MTs in CCP1 cKO medial ganglionic eminence (MGE) extracts, as detected by GT335 and PolyE antibodies, recognizing either the branching point glutamate or linear, carboxy-terminal glutamate stretches containing at least 3 glutamate amino acids (3E+) (Rogowski et al., 2010), respectively (Figures 1F and 1G; percentage of change, GT335: +87%, p = 0.035; PolyE: +127.1%, p = 0.0025). These results were further confirmed by immunolabeling of CCP1 cKO or wild-type (WT) cINs (Figure 1H).

Time-lapse recordings were performed on MGE explants from E13.5 CCP1 WT or cKO (Movie S1), cultured for 1 day *in vitro* (DIV) on mixed cortical feeder. While we did not observe defects of growth cone splitting or differences in the numbers and length of neurites (Figures S1C–S1E), migrating CCP1 cKO cINs extended secondary neurites with longer life duration (Figures 1I and 1J; percentage of change, +40%, p < 0.0001). This phenotype was correlated with accumulation of acetylated tubulin, which marks long-lived MTs (Figures 1K and 1L; percentage of change, +41%, p = 0.029). The migration of cINs analyzed by tracking nucleokinesis in organotypic slices (Figure 1M), revealed that cINs from both genotypes move at comparable average speed (Figure 1N) and with similar nucleokinesis frequency (Figure S1F), but of shorter amplitude (Figure 1O, percentage of change, −13%, p = 0.0015). In addition, nuclei of CCP1 cKO cINs spend less time pausing (Figure 1P; percentage of change, −13%, p = 0.0011), thereby undergoing sliding movements (Figure 1M, red arrows indicate continuous nuclear movements that are not considered as nucleokinesis events). Position of the centroid of a migrating cIN varies with time because it constantly grows and retracts processes during migration. We plotted the centroid displacement over time and found that, similar to the nucleus behavior, it pauses less frequently upon *Ccp1* knockdown (Figures S1G–S1I). Altogether, these data suggest that CCP1 is a pivotal enzyme expressed by cINs to control saltatory migration. Furthermore, overexpression of *Ccp1*, or its catalytic dead form, does not change the average speed (Figure S1J), the pausing time (Figure S1K) or the directionality (Figure S1L) of migrating cINs.

### CCP1 Regulates Actomyosin Dynamics

**Figure 2:**
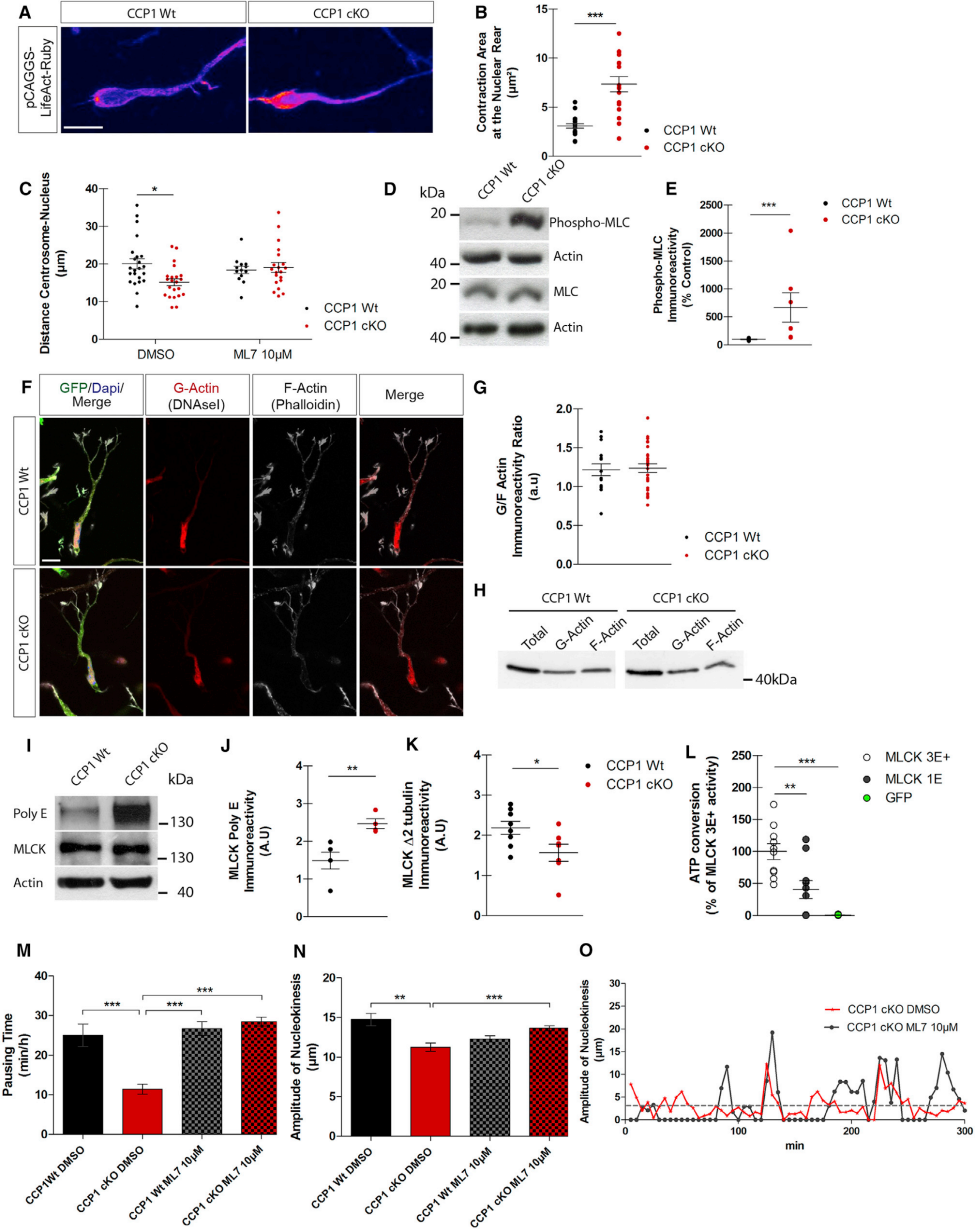
CCP1 Regulates Actomyosin Contraction by Processing MLCK (A) Co-culture of migrating cINs from CCP1 WT and cKO electroporated with pCAGGS-LifeAct-Ruby. Fire look-up table from ImageJ is applied to the image. Scale bar, 10 μm. (B) LifeAct-Ruby maximum intensity area at the nuclear rear during nucleokinesis in CCP1 WT and cKO (n = 16–19 cells in at least 3 embryos from at least 3 females; p < 0.001). (C) Maximal distance between the nuclear center and centrosome during nucleokinesis, in migrating cINs from CCP1 WT and cKO E13.5 embryos treated with 10 μM ML7 or DMSO (n = 13–23 cells from in at least 3 embryos from at least 3 females; p < 0.05). (D and E) MLC phosphorylation levels on protein extracts from E13.5. CCP1 WT and cKO GEs (D) and its quantification (E) normalized on actin and expressed as percentage of control, n = 7 embryos per group from 3 pregnant females. (F) Immunodetection of G actin (red) and F actin (white) on cultured cIN explants on Matrigel. cINs from CCP1 WT and cKO express GFP and DAPI^+^ nuclei. Scale bar, 10 μm. (G) Quantification of the G- and F-actin immunoreactivity represented in (F) and expressed as the ratio of G fraction/F fraction (n = 15–26 embryos from at least 3 females; parametric t test, p > 0.05). (H) Amount of G- and F-actin fractions purified from E13.5 CCP1 WT and cKO GEs. (I) Poly E, MLCK, and actin immunoreactivity on protein extracts from E13.5 CCP1 WT and cKO GEs. (J and K) ELISA quantification of Poly E (J) and Δ2-tubulin (K) immunoreactivity on MLCK in protein extracts from E13.5 CCP1 WT and cKO GEs. Luminescence levels are normalized on MLCK levels, n = 4–8 samples, non-parametric t test, p < 0.05. (L) MLCK activity in luminescence-based assay on ELISA purified GFP-MLCK. GFP-MLCK 3E^+^ and MLCK 1E as well as GFP were extracted from HEK293T cells, transfected with pCAGGS-GFP-MLCK 3E^+^, pCAGGS-GFP-MLCK 1E or pCAGGS-GFP. Normalized luminescence levels are expressed as percentage of MLCK 3E^+^ activity, n = 5–10 samples, one-way ANOVA, ^∗∗^p < 0.01, ^∗∗∗^p < 0.001. (M and N) Nuclear pausing time (M) and amplitude of nucleokinesis (N) of cINs from E13.5 CCP1 WT and cKO cINs migrating from MGE explants, n = 6–38 cells from at least 3 independent cultures, one-way ANOVA, ^∗∗^p < 0.01, ^∗∗∗^p < 0.001. (O) Example of the nuclear displacement of representative cINs from CCP1 cKO embryo treated with DMSO or ML7. The total time of displacement is 5 hr and every displacement above 5 μm (gray dotted line) is considered as a nucleokinesis. All graphs contain bars representing SEM. See also Figure S2.

**Figure S2:**
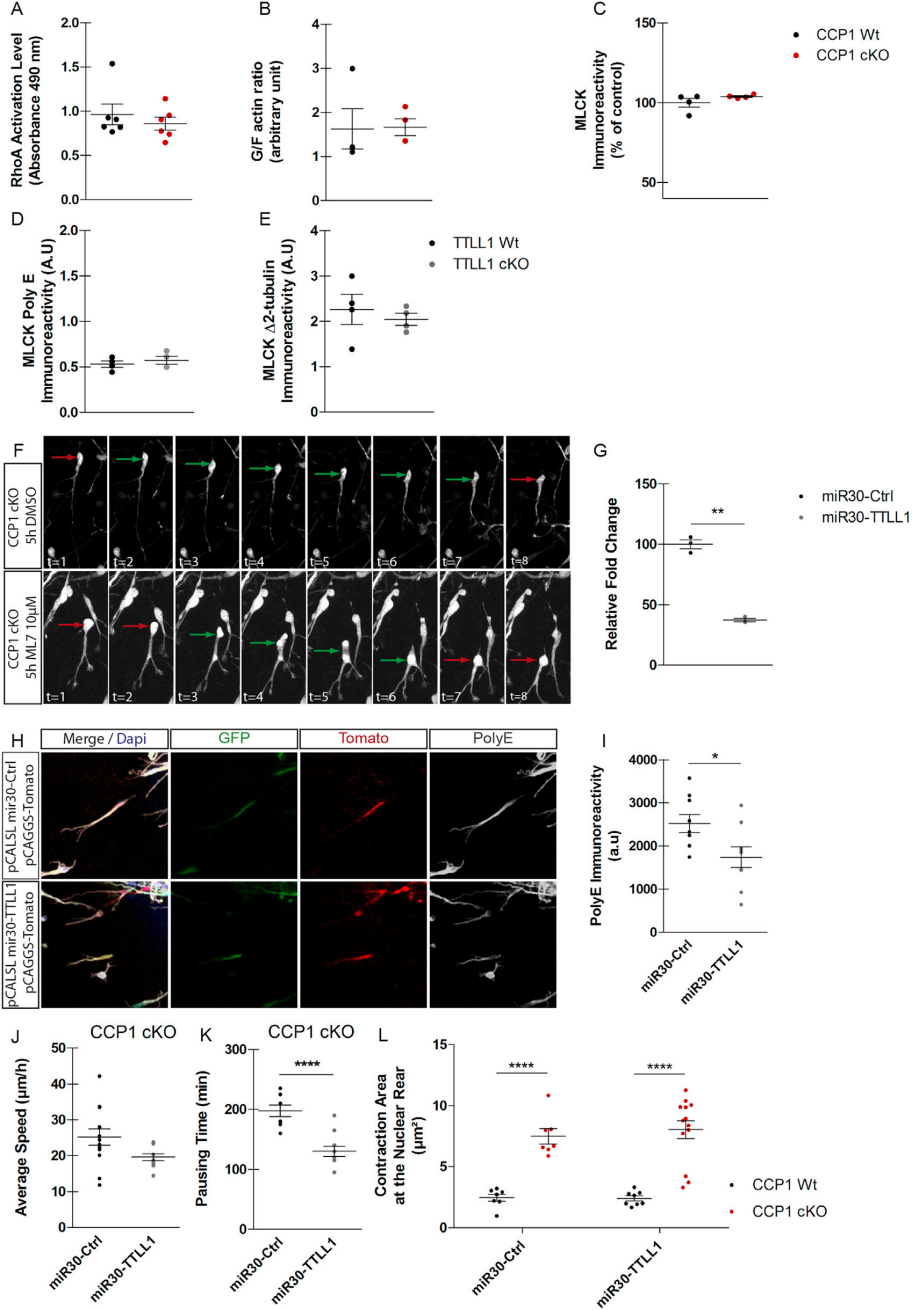
Cortical Interneurons Lacking *Ccp1* Show Nucleokinesis Defects Independent that Cannot Be Rescued by Blocking TTLL1 Activity, Related to Figure 2 (A) ELISA quantification of RhoA-GTP levels in E13.5 CCP1 WT and CCP1 cKO GEs protein extracts, n = 6 samples, non-parametric t test. (B) Quantification of the western blot of levels of globular (G) versus filamentous (F) actin in E13.5 CCP1 WT and CCP1 cKO GEs protein extracts, n = 4 samples, non-parametric t test. (C) Quantification of MLCK immunoreactivity levels detected by western blotting on E13.5 CCP1 WT and CCP1 cKO GEs protein extracts, n = 4 samples, non-parametric t test. (D and E) Quantification of MLCK-3E+ (Poly E antibody) (D) and MLCK-1E (Δ2-tubulin antibody) (E) immunoreactivity levels in sandwich ELISA in E13.5 TTLL1 WT and TTLL1 cKO GEs protein extracts, n = 4 samples, non-parametric t test. Immuno-luminescence is normalized on MLCK levels. (F) Time series of the nuclear displacement of cINs from E13.5 CCP1 cKO treated with DMSO (upper panels) and ML7 10 μM (lower panels). Green arrows indicate periods of nuclear movements and red arrows indicate periods of nuclear pausing. (G) Quantification by qRT-PCR of the efficiency of *TTLL1* mRNAs knock down in N2A cells transfected with pCALSL-mir30 TTLL1 and pCALSL-mir30 Ctrl, n = 3 cultures, non-parametric t test, p < 0.01. (H and I) Immunodetection (H) and quantification (I) of the polyglutamylation levels (Poly E antibody staining, white) in cINs co-electroporated with pCALSL-mir30 TTLL1 or pCALSL-mir30 Ctrl and pCAGGS-Tomato, n = 9 cells from at least 3 independent cultures, parametric t test, p < 0.05. (J and K) Quantification of speed of migration (J), and pausing time (K) of cINs co-electroporated with pCALSL-mir30 TTLL1 or pCALSL-mir30 Ctrl. (L) Quantification of LifeAct-Ruby maximum intensity area at the rear of the nucleus during nucleokinesis in time-lapse recording of cINs co-electroporated with pCALSL-mir30 TTLL1 or pCALSL-mir30 Ctrl and pCAGGS LifeAct-Ruby, n-9-13 cells from 3 independent cultures, parametric t test, p < 0.0001. All graphs contain bars representing SEM.

While migrating, CCP1 cKO cINs show minor impairment of MT-dependent neurite stability but exhibit dramatic nucleokinesis defects. Although (de)glutamylation has been mostly associated with MTs, it is now recognized that deglutamylase-sensitive C-terminal gene-encoded glutamate chains are present on proteins with function ranging from intracellular signaling to cytoskeleton regulation. The nucleokinesis of cINs mostly relies on actomyosin contractility that propels their nucleus forward to pace migration. Despite minor alteration of second order branch lifetime (Figures 1I and 1J), the nucleokinesis defects of CCP1 cKO cINs likely arise from poor regulation of actomyosin contraction. To test this hypothesis, we monitored actin dynamics during migration by expressing LifeAct-Ruby in cINs. In cINs these regions are the nuclear rear, the swelling preceding nucleokinesis, and the growth cone. We observed an enlargement of the contraction area behind the nucleus that adopted a cup-like shape in CCP1 cKO cINs (Figures 2A and 2B; Movie S2; percentage of change, +110%, p < 0.0001). Larger areas of actomyosin contraction result in less efficient nuclear movement due to dispersion of vector forces around the nucleus. Actomyosin contraction also controls centrosomal motility (Solecki et al., 2009), and the nucleus-to-centrosome distance of CCP1 cKO cINs was decreased (Figure 2C; Movie S3; percentage of change, −26%, p = 0.0373). Altogether, these differences support actomyosin dynamic defects upon loss of CCP1 in cINs. The subcellular localization of CCP1 in cINs coincides with regions of intense actomyosin remodeling (Figure 1D). Actomyosin contraction requires phosphorylation of the regulatory myosin light chain (MLC) (Somlyo and Somlyo, 2003). Indeed, the level of MLC phosphorylation is dramatically increased in CCP1 cKO cINs (Figures 2D and 2E; percentage of change, +568%, p = 0.0006). MLC phosphorylation is sustained through the RhoA pathway where ROCK kinase phosphorylates MLC directly (Amano et al., 1996) or indirectly by decreasing the activity of the myosin light chain phosphatase (MLCP) (Kimura et al., 1996). This pathway also controls indirectly actomyosin contractions by promoting the activity of LIM kinase, which further inhibits cofilin and thus prevents actin severing (Yang et al., 1998). Our analysis did not reveal difference in RhoA activation in E13.5 WT and CCP1 cKO cINs (Figure S2A). Moreover, protein levels of major actomyosin regulators remained unchanged between E13.5 CCP1 cKO or CCP1 WT MGE extracts (PRIDE: PXD006397). Analysis of globular (G) to filamentous (F) actin ratio suggested that the actin network was not altered in CCP1 cKO cINs (Figures 2F, 2G, and S2B). Another regulator of MLC phosphorylation is MLC kinase (MLCK), whose expression was similar in cINs of both genotypes (Figures 2I and S2C). We thus postulated that the modification of actomyosin contractility in CCP1 cKO cINs might originate from differences in MLCK activity rather than changes in expression.

### CCP1 Controls Pausing of cINs by Enzymatic Regulation of MLCK Activity

MLCK contains a C-terminal stretch of 8 glutamates that can be processed by CCPs *in vitro* (CCP1, CCP4, and CCP6) and give rise to a range of shorter forms of the protein (Rogowski et al., 2010). Because *Ccp1* is enriched in cINs (Figure 1B), we tested whether such processing would occur *in vivo*. For this purpose, MLCK western blot and pull-down from MGE extracts of E13.5 CCP1 cKO or CCP1 WT mice were analyzed with polyE antibodies to detect MLCK containing at least 3 glutamates (MLCK-3E^+^) or anti-Δ2-tubulin antibodies to detect MLCK-1E (Rogowski et al., 2010). We detected an increased polyE signal on MLCK immunoprecipitates from CCP1 cKO MGEs (Figures 2I and 2J, percentage of change, +133%, p = 0.0088), and a complementary reduced signal with Δ2-tubulin antibody (Figure 2K, percentage of change, −28%, p = 0.0424), suggesting accumulation of MLCK-3E^+^ at the expense of MLCK-1E. We next assessed whether MLCK was also processed by TTLL1, the predominant MTs glutamylase expressed by cINs (Figure 1B). MGE extracts of E13.5 TTLL1 cKO and WT were processed by ELISA. We detected equal distribution of signals for Δ2-tubulin and polyE immunoreactivity on MCLK immunoprecipitates, which strongly suggests that TTLL1 cannot glutamylate MLCK (Figures S2D and S2E).

To test whether MLCK processing by CCP1 alters its enzymatic activity, we performed *in vitro* activity assays of MLCK isolated from HEK293T expressing either MLCK-3E^+^ or MLCK-1E. After pull down, MLCK activity was measured by ATP consumption upon MRCL3 peptide phosphorylation. The kinase activity of MLCK-1E was 60% lower compared to the corresponding long form (Figure 2L, p = 0.0068). Therefore, by reducing MLCK activity via enzymatic processing and without affecting its expression in migrating cINs (Figure 2I; PRIDE: PXD006397), CCP1 might control nuclear movement during the stereotypic two-stroke cycle of migration. To test this hypothesis, we treated cultured MGE explants from CCP1 WT or CCP1 cKO cINs with ML7, a selective inhibitor of MLCK. ML7 treatment rescued pause duration (Figures 2M and S2F; Movie S4), amplitude of nucleokinesis (Figures 2N and 2O; Movie S4), as well as the maximum nucleus-centrosome distance in CCP1 cKO cINs (Figure 2C).

In the absence of CCP1 activity, several proteins accumulate long glutamate chains, most of them generated by glutamylases, which might modulate their function (Janke, 2014, van Dijk et al., 2008). To test if MLCK glutamylation status is a predominant regulator of cINs migration, we rescued general levels of protein glutamylation in CCP1 cKO cINs by expressing inducible small hairpin RNA (shRNA) that target *Ttll1* (Figure S2G). Acute reduction of TTLL1 in WT cINs decreased glutamylation levels (Figures S2H and S2I). Lowering *Ttll1* expression in cINs from E13.5 CCP1 cKO did not change their average speed of migration (Figure S2J) but worsen their migration phenotype by further reducing the pausing time (Figure S2K). Targeting *Ttll1* in a CCP1 cKO background did not rescue the size of actomyosin contraction area behind the nucleus (Figure S2L). Altogether, these results show that MLCK activity can be tuned by deglutamylation, however, the protein cannot be re-glutamylated by TTLL1, the major glutamylase expressed in interneurons (Figure 1B). Moreover, they suggest that conversion of the CCP1 cKO cINs migration mode is mainly resulting from dysregulation of MLCK activity, which depends on the length of its carboxy-terminal glutamate stretch.

### Cortical Invasiveness of the cINs Population Is Enhanced upon Deletion of Ccp1

**Figure 3:**
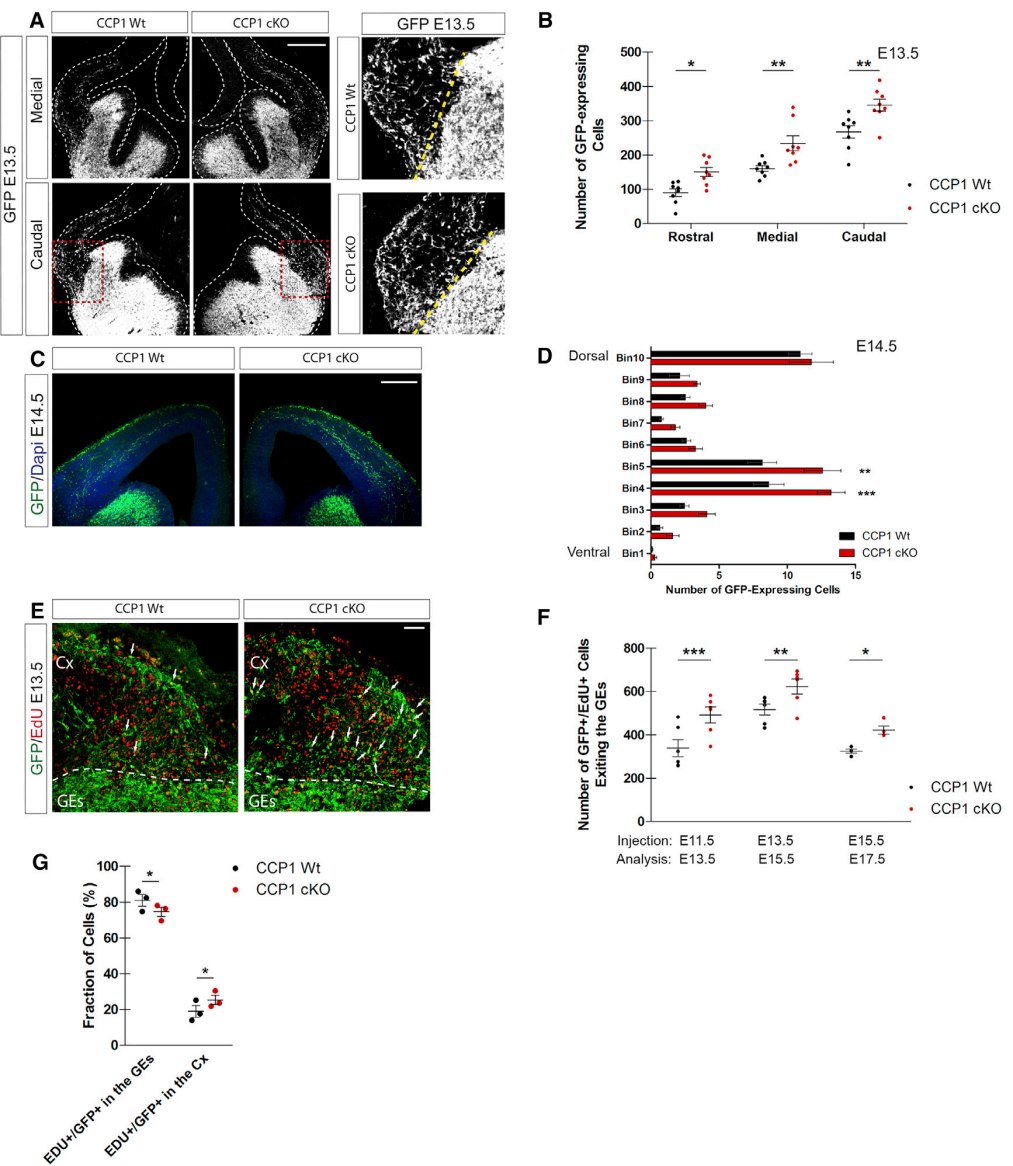
CCP1 Depletion Leads to Enhanced Cortical Invasion by the cIN Population (A) E13.5 brain sections from CCP1 WT and cKO embryos. cINs express Cre-GFP (white) and the contour of the brain is highlighted with dashed lines. Right: magnification of the CSB (yellow line). Scale bar, 100 μm. (B) Quantification of the cINs crossing the CSB at E13.5 in brain tissue from CCP1 WT and cKO, at different rostro-caudal levels, n = 8 embryos per group from at least 3 females, two-way ANOVA, ^∗^p < 0.05, ^∗∗^p < 0.01, ^∗∗∗^p < 0.001. (C) E14.5 brain sections from CCP1 WT and cKO embryos. cINs express Cre-GFP and nuclei are DAPI^+^. Scale bar, 100 μm. (D) Number of cINs in the cortex CCP1 WT and cKO embryos at E14.5. (E) EdU (red) immunodetection at the CSB in brain sections from CCP1 WT and cKO at E13.5, 48 hr post-injection. White arrows indicate EdU^+^ cINs. Scale bar, 50 μm. (F) Analysis of EdU^+^ cINs cohorts invading the cortical plate, genotypes as indicated. The time points of EdU injection and sacrifice were respectively: E11.5 to E13.5, E13.5 to E15.5, and E15.5 to E17.5 (n = 4–6 embryos from at least 3 females; two-way ANOVA, ^∗^p < 0.05, ^∗∗^p < 0.01, ^∗∗∗^p < 0.001). (G) Analysis of the position of the EdU-labeled cINs at E13.5, 48 hr after injection in CCP1 WT and cKO brain sections. EdU-labeled cINs are either found in the GEs or in the cortex (n = 3 embryos per group from 3 females; two-way ANOVA, ^∗^p < 0.05). All graphs contain bars representing SEM. See also Figures S3 and S4.

**Figure S3:**
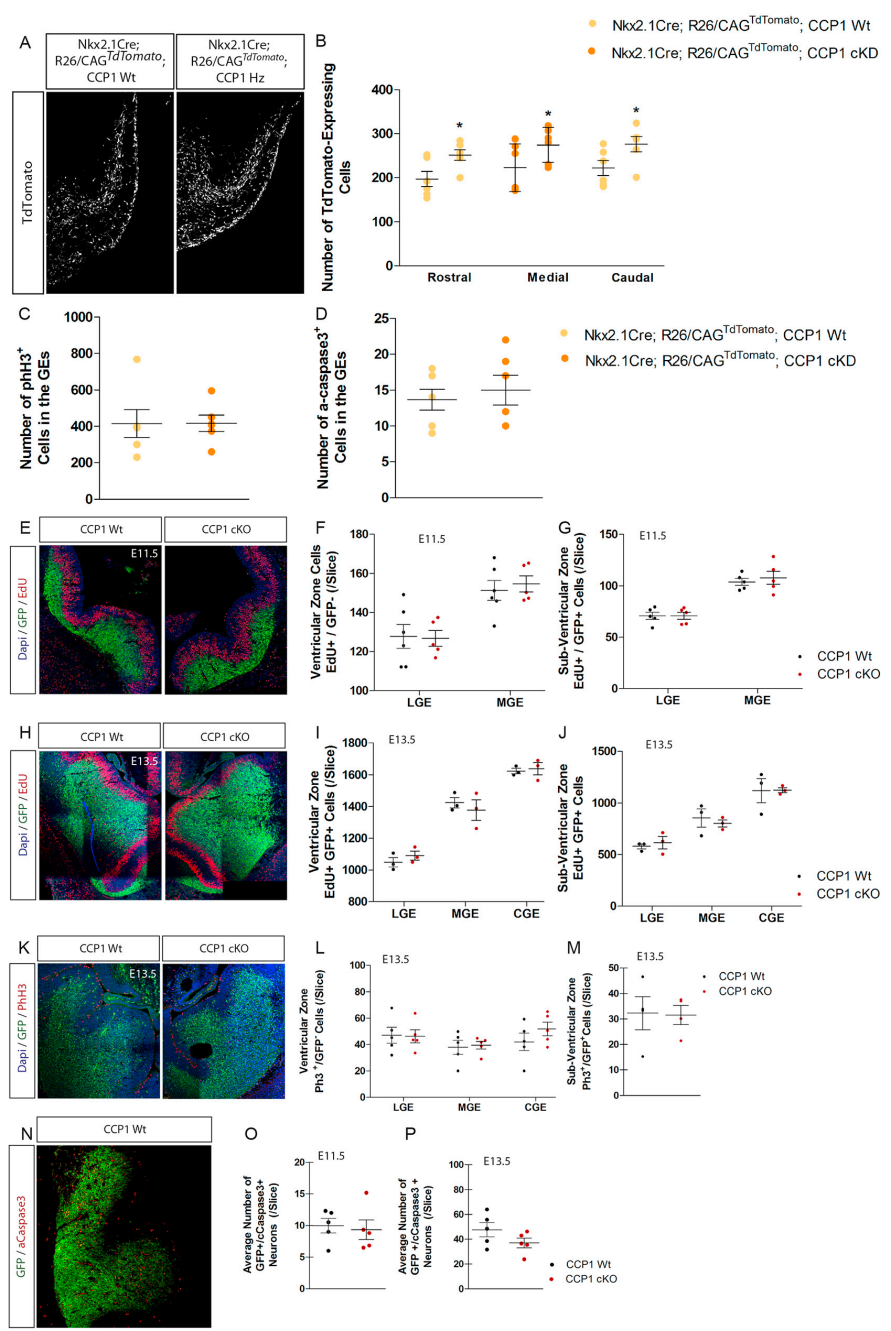
The Increased Cortical Invasion by CCP1 cKO Interneurons Does Not Result from Over Proliferation and/or Survival, Related to Figure 3 (A and B) Immunodetection (A) and quantification (B) of the numbers of TdTomato-expressing cells at different rostral to caudal levels in brain sections from E13.5 *Nkx2.1*Cre; *R26/CAG*^TdTomato^; CCP1 WT and Nkx2.1Cre; *R26/CAG*^TdTomato^; CCP1 cKD. n = 6 embryos from at least 3 independent female donors, two-way ANOVA, p < 0.05. (C and D) Quantification of the number PhH3^+^ cells (C) and activated caspase 3^+^ (D) cells in the ganglionic eminences of E13.5 *Nkx2.1*Cre; *R26/CAG*^TdTomato^; CCP1 WT and *Nkx2.1*Cre; *R26/CAG*^TdTomato^; CCP1 cKD. n = 6 embryos from at least 3 independent female donors, non-parametric t test. (E–J) Immunodetection of EdU (1h post-injection) and quantification of the number of S-phase cells in the VZ and SVZ of E11.5 (E)-(G) or E13.5 (H)-(J) of CCP1 WT and cKO embryos, n = 3-6 embryos from at least 3 independent females, two-way ANOVA. (K–M) Immunodetection of phospho-histone 3 (PhH3) (K) and quantification of the number of mitosis in the VZ (L) and SVZ (M) of E13.5 of CCP1 WT and cKO embryos. n = 4-5 embryos from at least 3 independent females, two-way ANOVA. (N–P) Immunodetection of activated caspase-3 (N) and quantification of the number of apoptotic cells in the GEs of E11.5 (O) and E13.5 (P) of CCP1 WT and cKO embryos, n = 5 embryos from at least 3 independent females, non-parametric t test. All graphs contain bars representing SEM.

We investigated how loss of *Ccp1* expression affects cIN migration *in vivo*. During development, cINs migrate from the subpallium to the pallium and by E13.5, some have already crossed the corticostriatal barrier (CSB) to enter into the developing cortex (Figure 3A). Magnified areas surrounding the CSB showed higher number of CCP1 cKO cINs invading the pallium at all rostro-caudal levels assessed (Figures 3A and 3B; percentage of change in rostral, +67%, p ≤ 0.05; medial, +45.4%, p ≤ 0.001; caudal, +29.1%, p ≤ 0.001). Bin analyses of the cortical wall of E14.5 mice showed accumulation of supernumerary cINs in the intermediate zone (IZ) migration stream (bins 4 and 5) of CCP1 cKO embryos (Figures 3C and 3D; percentage of change in bin 4, +52.9% p ≤ 0.001; bin 5, +54.1%, p ≤ 0.01). Comparable results were obtained when comparing E13.5 WT with *Ccp1* heterozygous embryos (CCP1 cKD; homozygous CCP1 embryos are not viable) arising from breeding between *Ccp1*^lox/lox^ mice and *Nkx2-1* Cre; R26/CAG^*TdTomato*^ mice (Fogarty et al., 2007) to recombine *Ccp1* allele in MGE cells (Figures S3A and S3B). While checking the origin of supernumerary cINs observed in the cortex of E13.5 CCP1 cKO (from *Dlx5-6*:Cre-GFP) and CCP1 cKD (from *Nkx2-1*-Cre) embryos, we found no impairment of progenitor proliferation rate (Figures S3C and S3E–S3M) or apoptosis (Figures S3D and S3N–S3P), which is frequent during development in the GEs (Southwell et al., 2012).

**Figure S4:**
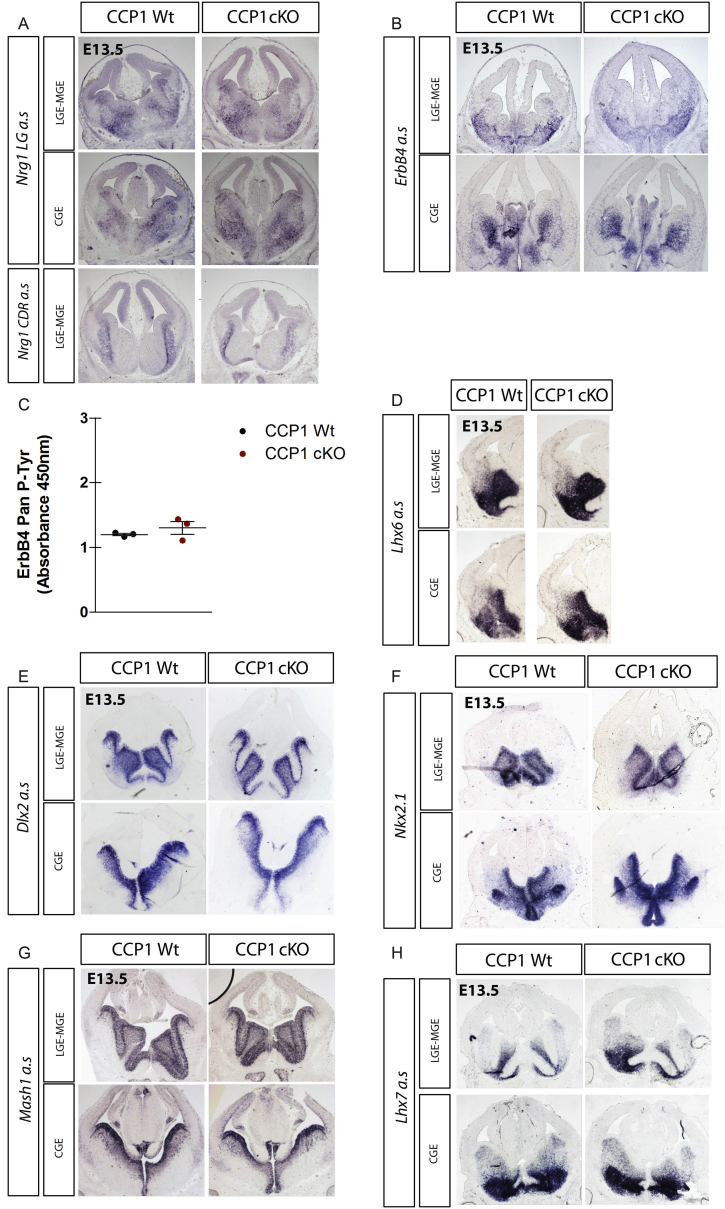
Analysis of the Expression Pattern of Chemoattractants and Cell-Fate-Specification Regulators in the Forebrain of CCP1 cKO and WT Embryos, Related to Figure 3 (A and B) *In situ* hybridization of *Nrg1 LG* and *CRD* (A) and *Erbb4* (B) in brain sections from E13.5 CCP1 WT and CCP1 cKO embryos. Medial (LGE-MGE) and Caudal (CGE) levels of the forebrain are shown. (C) ELISA quantification of the pan phosphorylation levels on Erbb4 receptor tyrosine residues in CCP1 WT and CCP1 cKO E13.5 GEs protein extracts, n = 3 samples, non-parametric t test. (D–H) *In situ* hybridization to detect *Lhx6* (D), *Dlx2* (E), *Nkx2.1* (F), *Mash1* (G), *Lhx7* (H) in cINs in brain sections from E13.5 CCP1 WT and CCP1 cKO embryos.

Attraction and guidance of cINs in the IZ rely on locally secreted type I neuregulin (Nrg1) that binds and activates the ErbB4 receptors expressed by cINs (Flames et al., 2004). Forebrain expression patterns of long range (LG) and cysteine-rich-domain (CDR) *Nrg1*, as well as *Erbb4* remained unchanged in E13.5 CCP1 cKO mice (Figures S4A and S4B). ELISA quantification revealed no differences of ERBB4 phosphorylation between protein lysates from CCP1 cKO and CCP1 WT GEs (Figure S4C). Together, these results suggest no disruption of the forebrain NRG1-ERBB4 pathway upon CCP1 deletion in cINs.

**Figure S5:**
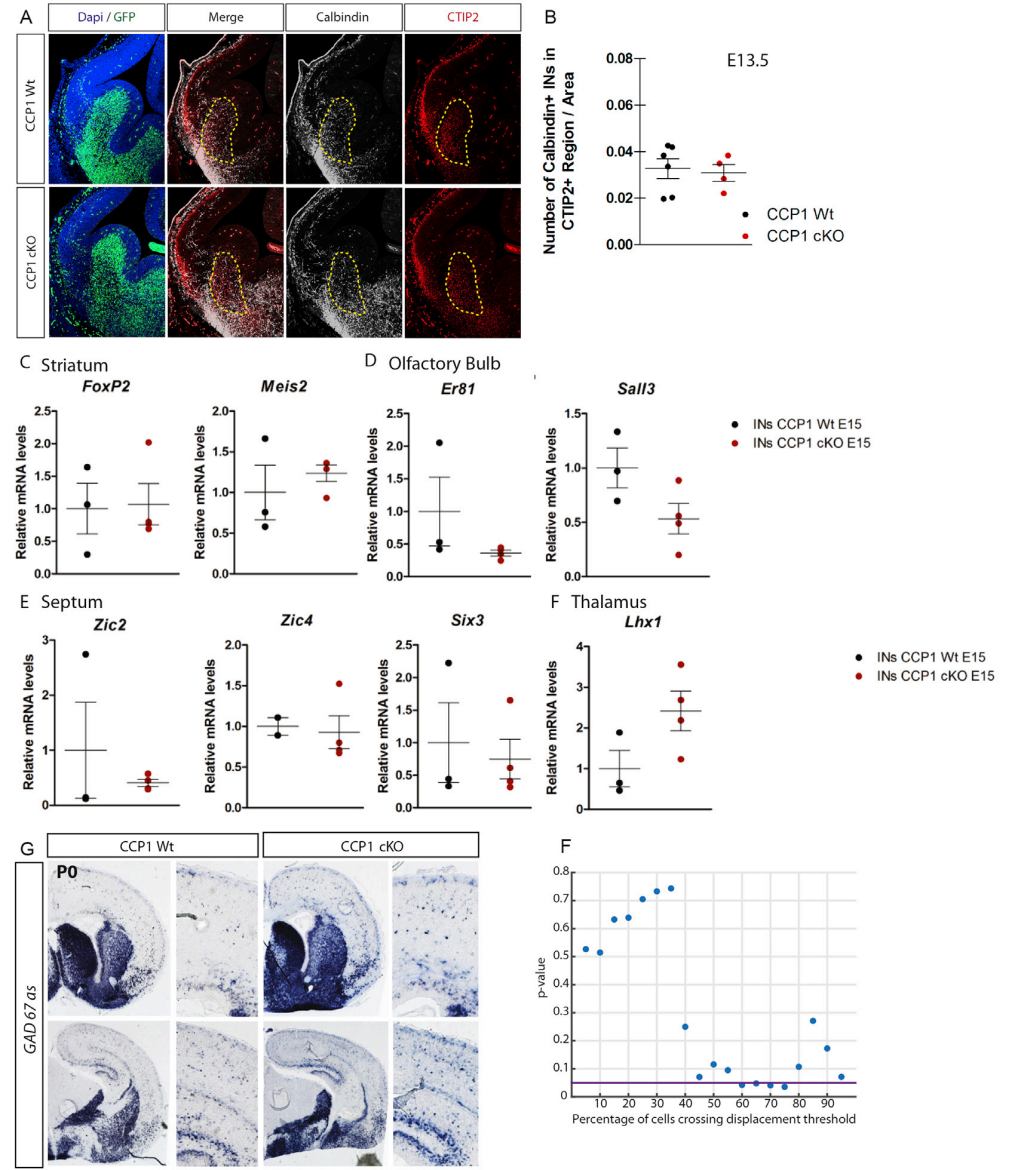
Analysis of the Interneuron Distribution in Distinct Regions of the Forebrain of CCP1 cKO and WT Embryos, Related to Figures 4 and 5 and Table S1 (A and B) Immunolabeling (A) and quantification (B) of the number of cINs (Calbindin, green and white) crossing the presumptive striatum region (Ctip2^+^, red) (delimited by a yellow dashed line) in brain sections from E13.5 CCP1 WT and CCP1 cKO embryos, n = 5-6 embryos from at least 3 independent female donors, non-parametric t test. (C–F) qRT-PCR analysis to compare level of expression of selected genes (as reported in the figure) expressed by FACS-purified cINs from E15.5 CCP1 WT or CCP1 cKO embryos, n = 3-4 samples, non-parametric t test. (G) *In situ* hybridization to detect *GAD67* on brain sections from P0 CCP1 WT and CCP1 cKO animals. (F) Statistical analysis of cell distributions showing the fraction of cells and observed difference. All graphs contain bars representing SEM.

In order to study the respective contribution of MGE and CGE to the supernumerary cINs invading the cortex of CCP1 cKO, we performed birth-dating analyses 48 hr after 5-ethynyl-2'-deoxyuridine (EdU) injection at different developmental stages (E11.5, E13.5, and E15.5). Higher numbers of EdU^+^;GFP^+^ cINs were observed at all rostro-caudal levels in the cortex of CCP1 cKO embryos (Figures 3E and 3F; percentage of change at E11.5–E13.5, +45%, p = 0.018; E13.5–E15.5, +20.6%, p = 0.028; E15.5–E17.5, +30%, p = 0.029). This suggests that supernumerary cINs in CCP1 cKO are generated from both MGE and CGE. We further confirmed that the expression pattern of core genes required for the specification and migration of cINs remained unchanged in CCP1 cKO embryos (Figures S4D–S4H). The MGE also produces striatal INs (sINs) whose sorting depends on cortical repulsion and striatal attraction (Marín et al., 2001). To assess whether supernumerary cINs seen upon *Ccp1* deletion might correspond to mis-positioned sINs, we quantified the density of GFP^+^; Calbindin^+^ cINs in CTIP2^+^ striatal regions and found no differences between genotypes (Figures S5A and S5B). Because additional brain regions are seeded by GABAergic neurons derived from *Dlx5-6*-expressing progenitors, we measured the expression level of genes selectively expressed by these neurons in fluorescence-activated cell sorting (FACS)-purified cINs from microdissected cortices of E15.5 CCP1 WT and CCP1 cKO. Our analysis revealed no differences of expression for these markers between genotypes (Figure S5C). FACS analyses revealed that the total number of GFP^+^ cells in the brain of E14.5 CCP1 cKO and WT embryos was comparable (±1.6 × 10^6^). Altogether, these results suggest that the increased number of cINs invading the cortex of CCP1 cKO embryos does not arise from differences of generation, survival, specification, or rerouting. While the total number of GFP^+^ cINs is similar between genotypes, the position of a cINs cohort born at E11.5 is more advanced in its journey at E13.5 in CCP1 cKO embryos as compared to controls (Figures 3E–3G). These data support that the increased flow of cINs invading the cerebral cortex upon loss of CCP1 activity might exclusively result from dysregulation of their migration.

**Figure 4:**
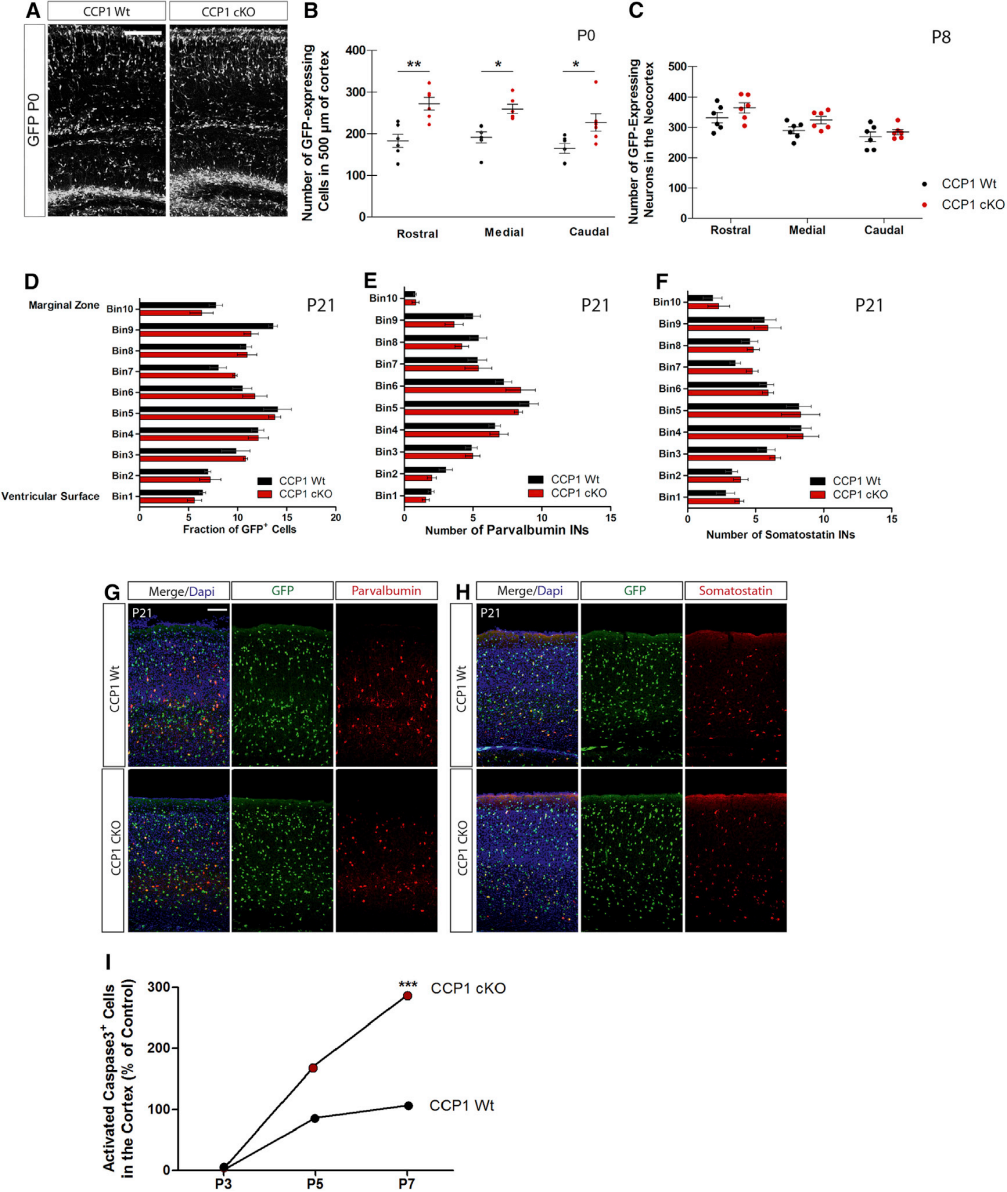
CCP1 cKO Cortical Invasion Phenotype Is Rescued after Birth (A) Brain sections of P0 CCP1 WT and cKO animals (GFP in white). (B and C) Number of cINs from CCP1 WT and cKO newborns at different rostro-caudal levels (n = 6 embryos from at least 3 females; two-way ANOVA, ^∗^p < 0.05, ^∗∗^p < 0.01, ^∗∗∗^p < 0.001). Scale bar, 500 μm. (B) Numbers of GFP^+^ cINs in rostro-caudal brain sections from CCP1 WT and cKO P8 pups (C). (D–F) Numbers of GFP^+^ (D), PV^+^ (E), or SOM^+^ (F) cINs, in rostro-caudal brain sections from P21 CCP1 WT and cKO animals (n = 3–6 mice; two-way ANOVA). (G and H) Immunolabeling showing PV^+^ (G) (red) or SOM^+^ (H) (red) expression in cINs (green) on brain sections from P21 CCP1 WT and cKO animals. Scale bar, 100 μm. (I) Quantification of the immunoreactivity of activated caspase 3 among GFP^+^ cINs in brain tissue from CCP1 WT and cKO P3, P5, and P7 pups (n = 3–6 embryos from at least 3 females; two-way ANOVA, p < 0.001). All graphs contain bars representing SEM. See also Figures S4 and S5.

Supernumerary cINs remain present in the cortex of newborn CCP1 cKO mice (Figures 4A, 4B, and S5G: percentage of change in rostral, +48.9%, p ≤ 0.01, medial, +35.6%, p ≤ 0.05, caudal, +37.8%, p ≤ 0.05) and later at P8 (Figure 4C). In order to assess whether the number and distribution of cIN subtypes (parvalbumin+, PV^+^ or somatostatin+, SOM^+^) was affected by loss of CCP1 activity in P21 animals, we permanently labeled cINs with YFP by crossing CCP1 cKO or WT (from the *Dlx5-6*-Cre-GFP background) with R26R-EYFP mice. Rostro-caudal binning of somatosensory cortex dorso-lateral areas, revealed no differences in total cIN numbers (Figure 4D) or number and laminar distribution of PV^+^ or SOM^+^ cINs (Figures 4E–4H). The first postnatal week, is characterized by an important wave of cell death regulating cIN number (Southwell et al., 2012). Quantification of cell death in the cortex of CCP1 cKO and WT mice at P3, P5, and P7 showed a progressive and significantly higher incidence of apoptosis in P7 CCP1 cKO cortices (Figure 4I). Together, these data suggest that the early overflow of CCP1 cKO cINs is later adjusted by increased rate of cortical apoptosis.

### Cell-Intrinsic Migration Properties Control the Rate of cINs Cortical Invasion

**Figure 5:**
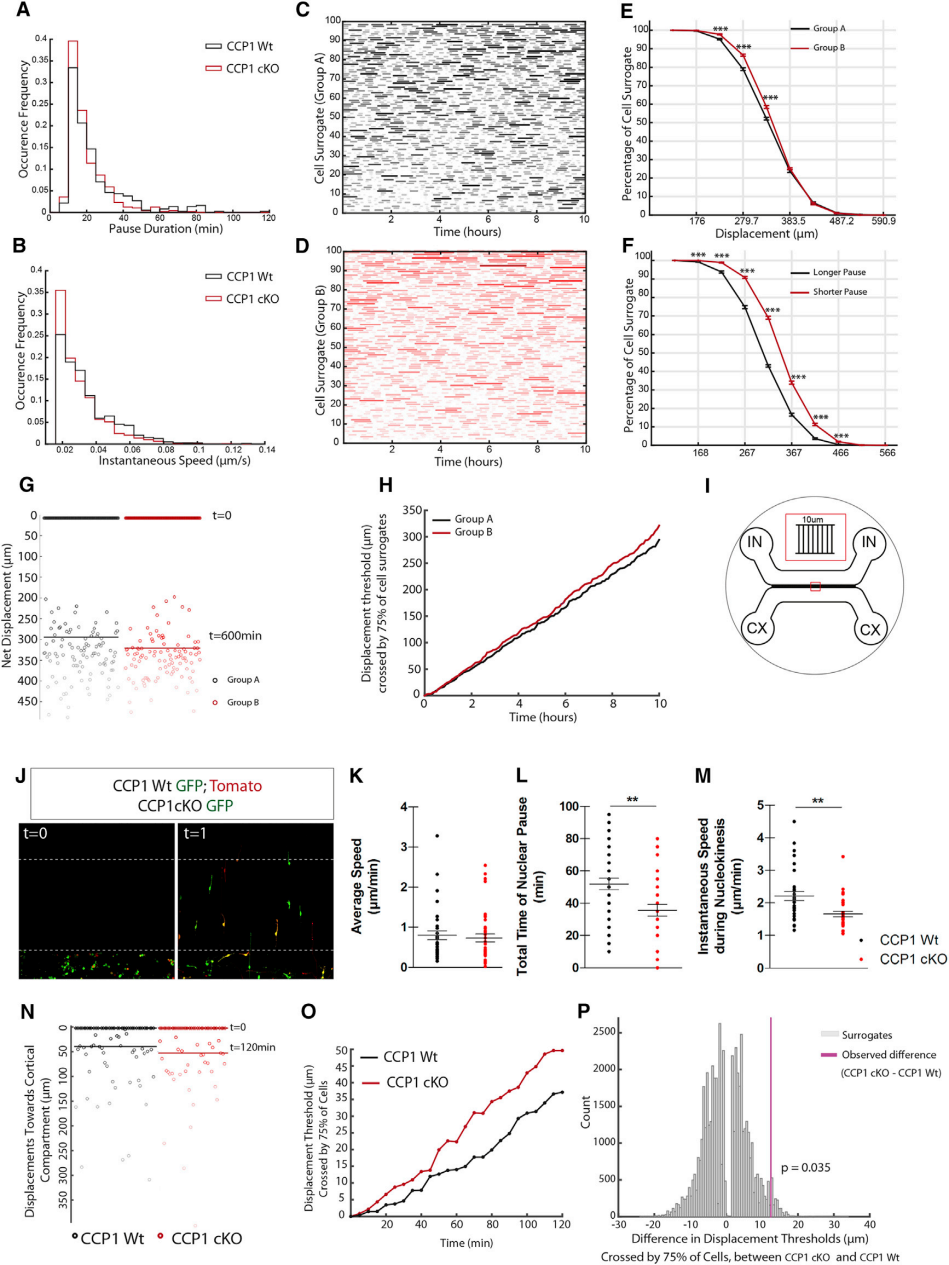
*In Silico* Modeling of cINs Migration Supports Enhanced Invasion of CCP1 cKO cINs (A and B) Frequencies of occurrence of pause duration (A) and instantaneous speeds (B) measured in organotypic slice cultures from CCP1 WT and cKO. (C and D) Movement phase densities (moving: colored, non-moving: white) for 100 cell surrogates of group A (C) and group B (D) as a function of simulated time, in one realization. Surrogates are ordered in the ascending order of total time spent in moving phase; darker shade indicates longer continuous time segments the surrogate spends in moving phase. (E) Percentage of cell surrogates that have crossed a displacement threshold, at the end of simulation time of 10 hr, as a function of displacements. The 10 displacement values are equally distributed between minimum and maximum displacements from both groups, n = 20 cells, unpaired t test, p < 0.001. (F) Same as (E) except instantaneous speeds of cell surrogates in both groups are sampled from the same distribution. Pause durations of groups A and B are sampled from distributions of pause durations of CCP1 WT and cKO, n = 20 cells, unpaired t test, p < 0.001. (G) Initial and final positions of all cell surrogates in both groups; lighter marker shade indicates higher total displacement of a surrogate. Horizontal bars mark the highest displacement thresholds crossed by 75% of surrogates in each group. (H) Highest displacement threshold crossed by 75% of cell surrogates of group B is greater than the corresponding value in group A at almost all time instances during simulation. (I) Representation of MFD. CX, cortical cell compartments; IN, cIN compartments. (J) Representative starting and end point of time-lapse acquisition of CCP1 WT; Rosa 26^Tomato^; Dlx5/6 CRE GFP and CCP1 cKO; Dlx5/6 CRE GFP mixed cINs population migrating in the corridors of the MFDs. (K–M) Average speed (K), time of nuclear pause (L), and amplitude of nucleokinesis (M) in CCP1 WT and cKO cINs population measured in 120-min time-lapse acquisition of migration in the MFDs, n = 34–41 cells from at least 3 embryos, unpaired t test, p < 0.01. (N) Displacement of CCP1 WT and cKO cINs populations. Horizontal bars mark the highest migration distance toward cortex crossed by 75% of all cells in each population. (O) Highest migration distance crossed by 75% of cells from CCP1 cKO population is greater than the corresponding value in CCP1 WT population at all time instances. (P) The observed difference (magenta) between highest migration distance crossed by 75% of CCP1 WT and cKO cells in comparison with the frequency of occurrence of corresponding values of difference between two surrogate populations formed by randomly permuting the cell memberships of CCP1 WT and cKO populations 10,000 times. All graphs contain bars representing SEM. See also Figure S5.

Loss of CCP1 activity in cINs converts their saltatory biphasic mode of migration into a steady monophasic migration. While cINs of both genotypes migrate at the same average speed, pause duration, and instantaneous speed were significantly reduced in migrating CCP1 cKO cINs (Figures 5A and 5B). To test whether differences of kinetic properties of migration lead to an increased sorting of CCP1 cKO cINs in the developing cortex, we generated trial surrogates of cellular motion using distributions of kinetic parameters (Figures 5A and 5B). We simulated 20 realizations of two groups, A and B, of hundred surrogates with kinetic parameters similar to those observed *in vitro* for CCP1 WT cINs and CCP1 cKO cINs, respectively (Movie S5). One such realization displays reduced pause durations in group B (Figure 5D) as compared to group A (Figure 5C). We found that at the end of a simulation time of 10 hr, a significantly higher percentage of group B surrogate cells had crossed the lower displacement thresholds compared to that found in group A, while there was no significant difference between the two groups for higher displacement thresholds (>350 μm). Preserving identical distributions of instantaneous speeds for groups A and B showed significant difference in the percentage of cells crossing all displacement thresholds (Figures 5E–5H). Therefore, these data-driven simulations demonstrate that the reduced pausing of CCP1 cKO cINs increases their recruitment at short distances from the starting location despite a compensatory effect of lower instantaneous speeds (Figures 5E and 5F).

*In vivo*, cINs migrate in streams in which they might interact with each other, thereby controlling potential collective migration. Such regulation would be exacerbated in CCP1 cKO embryos whose IZ stream is more populated by cINs. In order to disentangle the confounding influence of intercellular communication on the *in vivo* phenotype, we performed time-lapse analyses of isolated CCP1 WT and cKO cINs migrating individually in microfluidic devices (MFDs) (Figures 5I and 5J). Differences of migration properties between CCP1 cKO and CCP1 WT cINs, as reported *in situ*, were preserved (Figures 5K–5M). The displacement threshold crossed by 75% of CCP1 cKO cINs was consistently higher than the one of CCP1 WT cINs (Figures 5N and 5O) and was significantly higher (p = 0.035) after 2 hr of time-lapse recording (Figures 5P and S5F; Movie S6). These results support the model that individual changes in migration properties of CCP1 cKO cINs result in a collective change that determines how they invade the developing cortex as a population.

### Fine-Tuning of Cortical Invasion by cINs Controls Dorsal Cortical Neurogenesis

**Figure 6:**
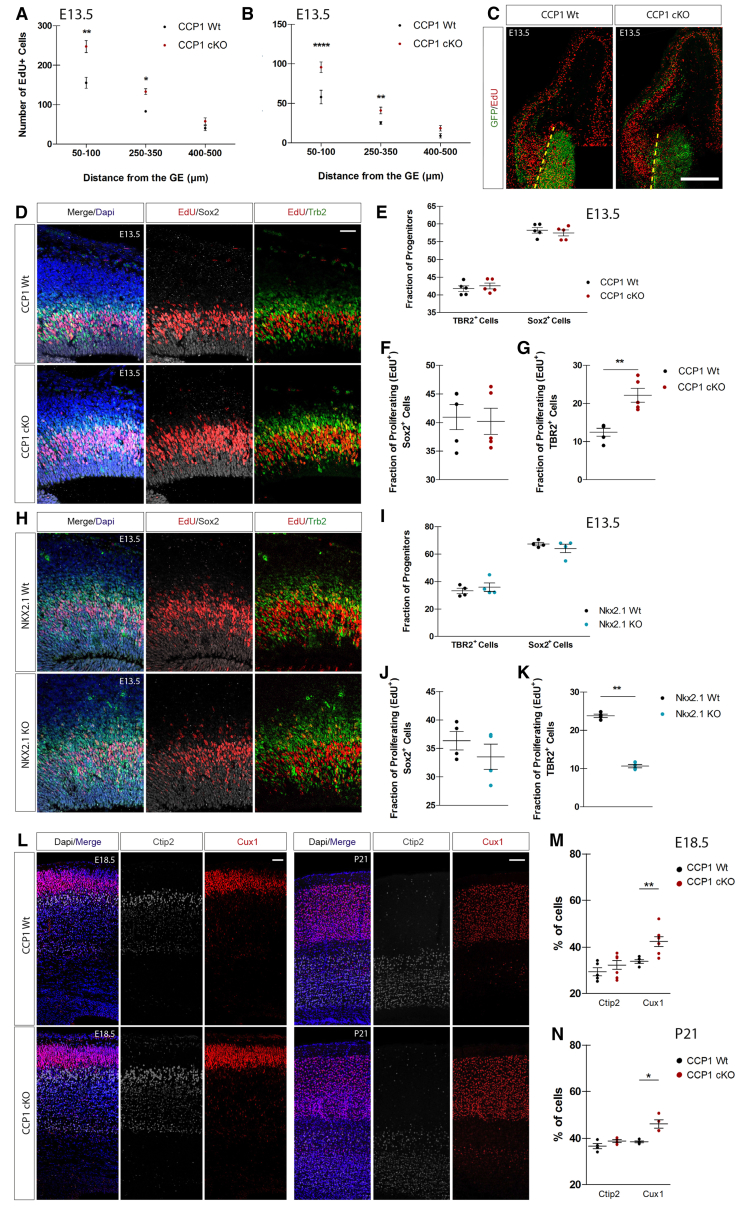
Bidirectional Molecular Communication between IPs and cINs Shapes Neocortex Histogenesis (A and B) Number of proliferating cells and cINs in the pallium (A), 1 hr post EdU injection at several distances from the CSB in CCP1 WT and cKO E13.5 brain sections (B) (n = 3 embryos from 3 females donors; two-way ANOVA, ^∗^p < 0.05, ^∗∗^p < 0.01, ^∗∗∗^p < 0.001). (C) EdU immunodetection 48 hr post-injection in E13.5 brain sections from CCP1 WT and cKO embryos. cINs express Cre-GFP and the CSB is highlighted by dashed yellow lines. Scale bar, 500 μm. (D) EdU, Sox2, and Tbr2 immunodetection on brain sections from E13.5 CCP1 WT and cKO embryos. cINs express Cre-GFP. (E) Number of Tbr2^+^ and Sox2^+^ normalized on total number of progenitor cells of the pallium in E13.5 brain sections from CCP1 WT and cKO embryos (n = 5 embryos from at least 3 females; non-parametric t test, p > 0.05). (F and G) Number of proliferating EdU^+^/Sox2^+^ (F) and EdU^+^/ Tbr2^+^ (G) in E13.5 brain sections from CCP1 WT and CCP1 cKO embryos, normalized on total number of progenitor cells of the pallium (n = 5 embryos from at least 3 females; non-parametric t test, p < 0.01). (H) EdU, Sox2, and Tbr2 immunodetection on brain sections from E13.5 Nkx2.1 WT and Nkx2.1 KO embryos. (I) Number of Tbr2^+^ and Sox2^+^ normalized on total number of pallial progenitor in E13.5 brain sections from Nkx2.1 WT and Nkx2.1 KO embryos (n = 4 embryos from at least 3 females; non parametric t test, p > 0.05). (J and K) Number of proliferating EdU^+^/Sox2^+^ (J) and EdU^+^/Tbr2^+^ (K) in E13.5 brain sections from Nkx2.1 WT and KO embryos, normalized on total number of progenitors (n = 4 embryos from at least 3 females; non-parametric t test, p < 0.01). Scale bar, 10 μm. (L) Immunodetection of Ctip2^+^ and Cux1^+^ at E18.5 and P21 on cortical sections from CCP1 WT and cKO brains. Nuclei are DAPI^+^. Scale bar, 100 μm. (M and N) Quantification in E18.5 (M) and P21 (N) CCP1 WT and cKO cortex, of the number of Ctip2^+^ and Cux1^+^ neurons in the cortical layers normalized on DAPI cell in the cortical plate (n = 4–8 embryos/adolescent mice; non-parametric t test, p < 0.01). All graphs contain bars representing SEM.

**Figure S6:**
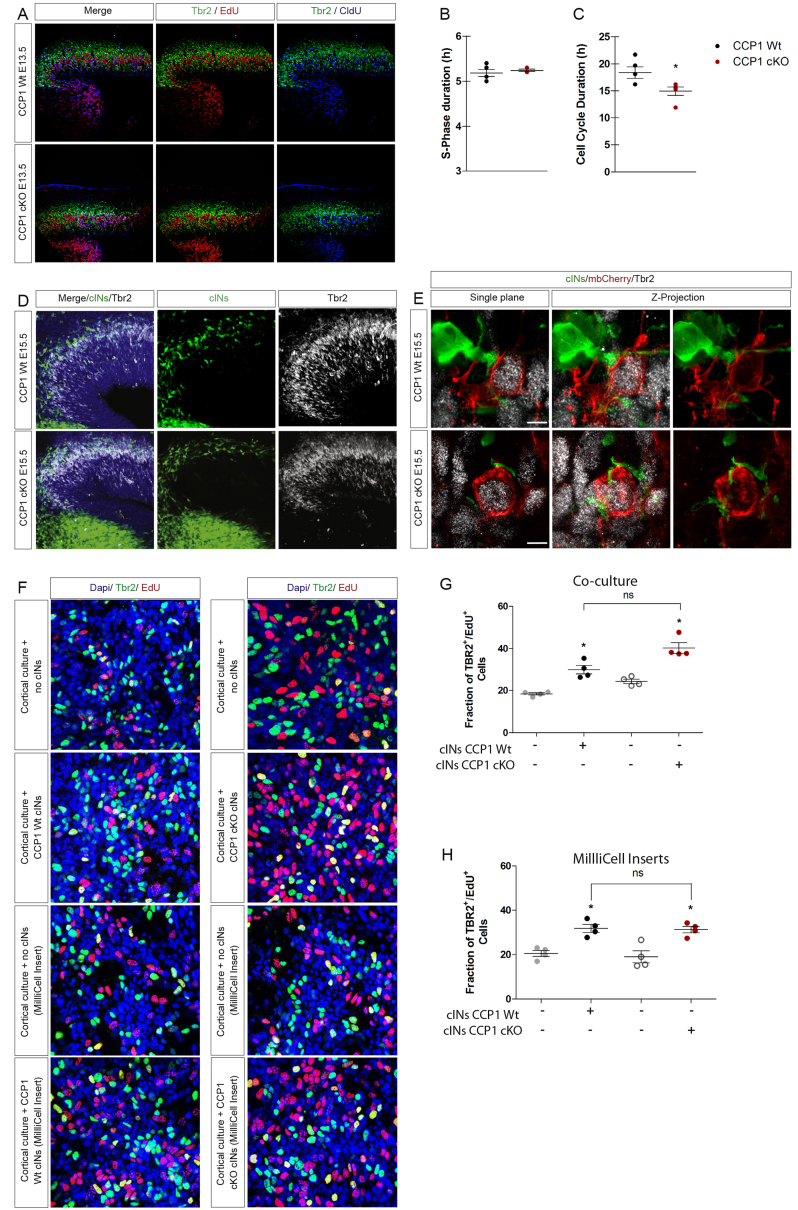
Quantification of the Cell-Cycle Parameters of Cortical Progenitors and Characterization of Interaction between Cortical Interneurons and Dorsal Intermediate Progenitors, Related to Figure 6 (A) Immunolabelings on brain slices from E13.5 CCP1 WT and CCP1 cKO embryos to detect injected CldU (blue) and EdU (red) in Tbr2^+^ cells (green). (B and C) Quantification of the S-phase (B) or total cell cycle duration (C) of Tbr2^+^ cells from CCP1 WT and CCP1 cKO at E13.5, n = 5 embryos from at least 3 independent females, non-parametric t test. (D) Immunolabelings on brain sections from E13.5 CCP1 WT and CCP1 cKO showing the spatial distribution of migrating cINs (green) and Tbr2^+^ IPs (white) at the subpallila-pallial boundary. (E) Single plane and Z-projection images showing the apposition between cINs (green) and Tbr2^+^ IPs expressing mbCherry (red membrane and white nuclear) at E15.5. (F) Immunolabelings of cortical progenitor cultures enriched in IPs (Tbr2^+^, green) undergoing S-phase (EdU^+^, red), nuclei are coutertsaind with Dapi (blue) with or without explants of MGE-derived-cINs isolated from CCP1 WT or CCP1 cKO E13.5 embryos. cINs were cultured with cortical progenitors (co-culture) or seeded in millicell inserts placed on top of cortical progenitors. (G) Fraction of cycling Tbr2^+^ IPs in co-culture with CCP1 WT or CCP1 cKO cINs, n = 4 cultures, two-way ANOVA, p < 0.05. (H) Fraction of cycling Tbr2^+^ IPs in the presence of millcell cultured cINs from CCP1 WT or CCP1 cKO E13.5 embryos, n = 4 cultures, two-way ANOVA, p < 0.05. All graphs contain bars representing SEM.

We have shown that the invasion of the cortical wall by cINs is modulated by changes in cell-intrinsic migration properties during development. However, the physiological role of a regulated sorting of cINs in the developing cortex remains unknown. To uncover such potential role, we analyzed the cellular properties of the cortical wall during its invasion by cINs. The neuronal cell output of dorsal progenitors was analyzed 48 hr after EdU injection, revealing a higher number of EdU^+^;GFP^−^ cells in E13.5 CCP1 cKO cortices (Figures 6A and 6C). We observed a strong correlation between numbers of EdU^+^; GFP^−^ PNs and cINs present in selected cortical regions (Figures 6B and 6C). The Pearson correlation coefficient was significant between these two parameters (r = 0.9397; p = 0.0002), showing that cortical regions containing a higher density of cINs have higher proliferation rates. We next assessed the fraction of cycling Sox2^+^ apical progenitors (APs) and Tbr2^+^ intermediate progenitors (IPs) located close to the CSB where cINs migrate. The proportions of APs and IPs were similar between genotypes (Figures 6D and 6E) but only the rate of IPs undergoing S-phase (1-hr EdU pulse) was increased in the cortical wall of E13.5 CCP1 cKO (Figures 6D, 6F, and 6G). We next performed a sequential double injection of thymidine analogs, CldU and EdU at E13.5, which demonstrated that while the cell-cycle length (Tc) of IPs was decreased, no change in S-phase duration (Ts) occurred upon loss of CCP1 activity (Figures S6A–S6C). To test whether the cycling behavior of IPs was sensitive to a reduction of cortical invasion by cINs, we monitored these parameters in *Nkx2-1* knockout (KO) mice that suffer from strong reduction of migrating cINs in the cortex (Butt et al., 2008). The analysis of cycling parameters of E13.5 *Nkx2-1* KO and WT mice revealed a selective reduction of IPs undergoing S-phase (Tbr2^+^; EdU^+^) (Figures 6H–6K). Cortical IPs populate the upper VZ and lower IZ where we observed many supernumerary cINs migrating in CCP1 cKO embryos (Figures 3C, 3D, and S6D). To assess the possible contact between both cell population, we performed super-resolution analyses of brain sections from E15.5 CCP1 WT and CCP1 cKO embryos dorsally electroporated with mbCherry. We observed a close apposition of cIN processes and the membrane of some Tbr2^+^ IPs (Figure S6E). In order to decipher whether a cellular contact between cINs and IPs is required to control their proliferation, we designed *in vitro* assays. Cortical progenitor cultures enriched in IPs were maintained 1DIV with or without homochronic explants of MGE-derived cINs (Figure S6F). The explants were either seeded on the cortical progenitor cultures (co-culture) or in Millicell inserts to avoid direct contact. Proliferation of IPs was measured after a short pulse of EdU (30 min). While proliferation of Sox2^+^ APs was similar across culture conditions (data not shown), we observed a larger fraction of cycling Tbr2^+^ IPs in cultures containing cINs explants. Importantly, direct contact was not required to stimulate IPs proliferation (Figures S6F–S6H; percentage of change of proliferation in co-cultures: cINs CCP1 WT +62%, cINs CCP1 cKO +66%; in Millicel inserts: cINs CCP1 WT +55%, cINs CCP1 cKO +64%). Interestingly, cINs from CCP1 WT embryos or CCP1 cKO embryos show the same ability to promote IP proliferation. These data suggest that cINs modulate Tbr2^+^ IPs proliferation paracrine signals.

In E13.5 CCP1 cKO mice, the proportion of cycling IPs is increased over control and this is correlated with increased cell output. At E13.5, cortical progenitors are mainly generating neurons for superficial layers (Hevner et al., 2003). Accordingly, we observed an increased number of Cux1^+^ neurons in the cortex of E18.5 CCP1 cKO embryos while the number of Ctip2^+^ neurons, generated before E13.5, remained unchanged across experimental groups (Figures 6L and 6M). Supernumerary Cux1^+^ PNs remained visible at P21, suggesting a permanent disruption of the cINs/PNs balance in CCP1 cKO cortices (Figures 6L and 6N).

**Figure 7:**
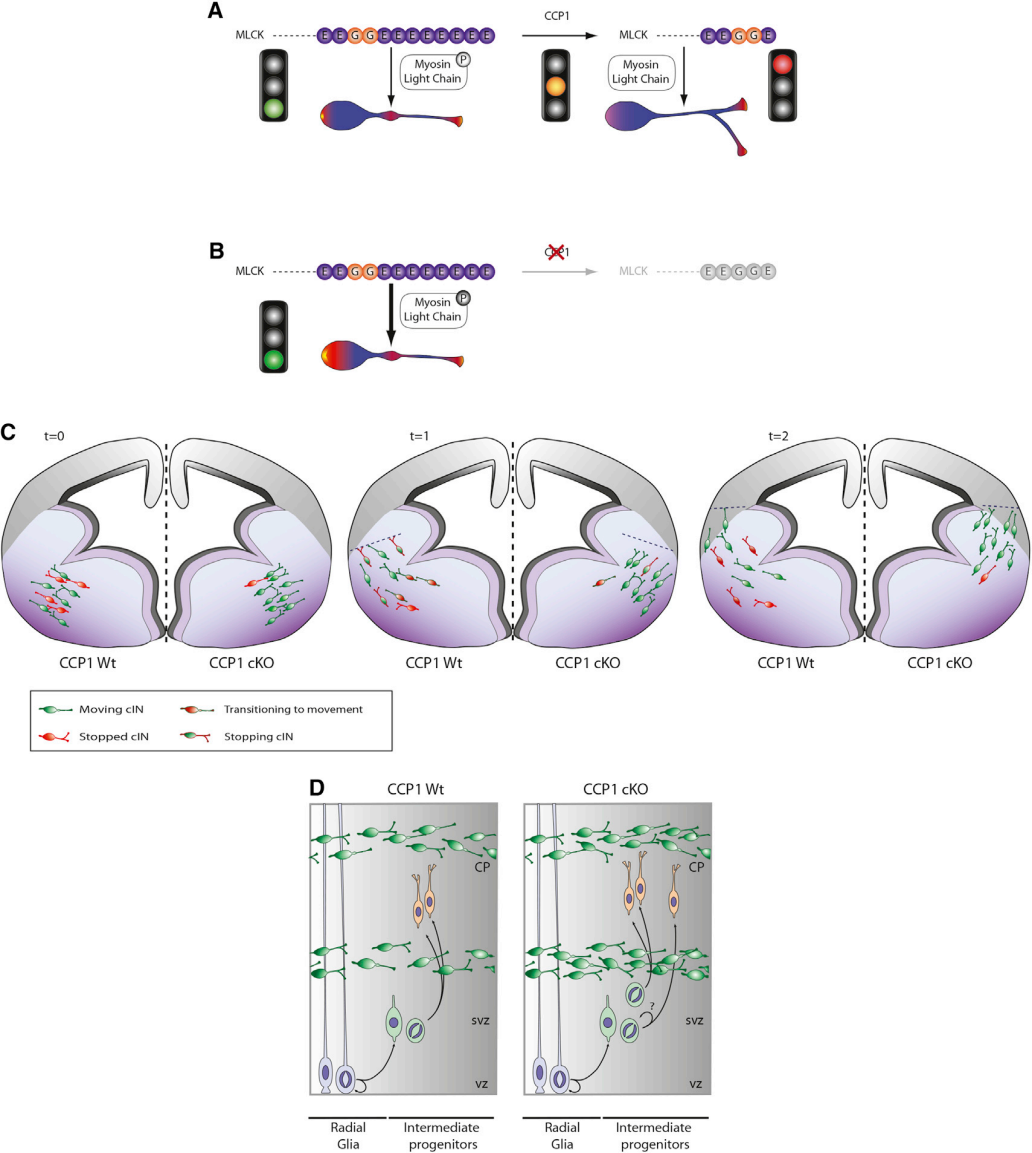
Role of CCP1 during cIN Migration in the Cerebral Cortex and Fine-Tuning of Dorsal Neurogenesis (A and B) MLCK processing by CCP1 in CCP1 WT (A) and cKO (B) and its effect on actomyosin contraction and cINs movement. (C) Conversion of cINs migration behavior modifies cortical invasion. (D) Changes in cortical invasion modify the proliferation of dorsal intermediate progenitors.

Altogether, these results suggest the existence of a crosstalk between IPs and cINs to regulate IPs proliferation, thereby the number of upper layer PNs. Therefore, neuronal output at mid-corticogenesis is modulated by the number of cINs invading the developing cortical wall, which itself is under the control of cell-intrinsic migration properties through MLCK regulation by CCP1 (Figures 7A–7D).

## Discussion

### Pausing during Migration Strongly Influences the Rate of Cortical Invasion by Interneurons

Pause duration and instantaneous migration speed of cINs can have contrary influences on the size of their cohort invading the cortex (Figures 5E and 5F). Moreover, the cINs recorded *in vitro* do not display strict movement linearity. Therefore, we used synthetic biology to characterize the cINs cohort displacement. For this purpose, we performed simulations by imposing movement linearity to surrogates, using the experimental distributions of cINs migration parameters and found a higher percentage of cells arriving to destination upon conditional loss of CCP1 activity. These simulations assumed a single direction of migration and no interaction between cells. Time-lapse analyses of isolated CCP1 WT and CCP1 cKO cINs migrating individually in MFDs tested both assumptions and validated the cell-intrinsic contribution to the migration of a cohort of cINs. These experiments also gave a closer picture to the *in vivo* situation. These results therefore provide significant evidence to conclude that increase in the size of the CCP1 cKO cINs cohort reaching the cortical wall is a result of the decreased pause duration, in spite of the compensatory effect of reduced instantaneous speeds and is not dependent on intercellular interaction.

### Deglutamylation Is a Novel Mode of Regulation of Enzymatic Activity

Despite the requirement of MT modifications to control INs migration and the existing subtle neurite branching defects upon accumulation of polyglutamylation, we show here loss of CCP1 activity in cINs reduces their pausing and instant migration speed by affecting mostly actomyosin contractions. By removing gene-encoded carboxy-terminal glutamates from MLCK, CCP1 regulates the enzymatic activity of MLCK. As such, our work demonstrates that the enzymatic activity of a protein can be directly regulated by enzymatic deglutamylation. Alterations in the carboxy-terminal glutamate stretch of MLCK might affect its folding or/and control its ATP loading efficiency or its ability to bind to MLC. It will be exciting to decipher the mechanism that control CCP1 activity, thus allowing it to timely control the pausing of migrating cINs. Our data show that CCP1 is detected both in the cytoplasm and in the nucleus of cINs (Figure 1D). Therefore, one possible explanation could be the existence of a regulated nucleo-cytoplasmic shuttling of CCP1 to pace intermittent periods of movements via cytoplasmic regulation of actomyosin contractions. Our future work will focus on the analysis of the regulation of the spatiotemporal activity of CCP1 activity in migrating cINs.

It is noteworthy that although cINs accumulate high levels of MT glutamylation that can be processed by de/glutamylases, the migration phenotype of CCP1 cKO cINs predominantly arises from the dysregulation of actomyosin contractions.

### Molecular Crosstalk between cINs and IPs Controls Cerebral Cortex Neurogenesis

Crosstalk between cINs and PNs is important to control early steps of cortical morphogenesis (Bortone and Polleux, 2009). Along this line, our work provides a physiological rationale for the transient pausing of migrating cINs in the cerebral cortex. Pausing not only regulates the flow of cINs invading the cortex by imposing movement heterogeneity at the population level, but also controls the proliferation of IPs for the generation of *ad hoc* number of upper layer PNs. While supernumerary cINs promote IPs proliferation, massive depletion of cINs invading the cortex of *Nkx2.1* KO mouse embryos leads to an opposite phenotype. However, *Nkx2-1* expression is also required for forebrain angiogenesis (Vasudevan et al., 2008), a factor regulating proliferation of cortical progenitors, including IPs (Javaherian and Kriegstein, 2009, Lange et al., 2016). In addition, *Nkx2.1* KO embryos lack some ventral forebrain structures (Sussel et al., 1999) such as MGE and CGE that are major sources of SHH, which modulate cortical progenitor proliferation (Komada et al., 2008). Our *in vitro* data suggest, however, that IPs proliferation can be modulated by the number of cINs, independently of vessels or integrity of ventral forebrain structures.

A balance between numbers of PNs and cINs settling into the cortex is primordial to ensure proper activity of the cortex, but its regulation and the exact nature of the molecular dialog existing between migrating cINs and IPs remains to be uncovered. Such dialog likely includes diffusible factors released form cINs that influence the cell-cycle progression of IPs, as suggested by our *in vitro* experiments.

IPs have previously been shown to regulate cINs migration via chemokine signaling (Sessa et al., 2010, Tiveron et al., 2006). Interestingly, the supernumerary cINs observed in the cortex of CCP1 cKO embryos were accumulating in the IZ migration stream, in close the vicinity of cycling IPs. Therefore, our finding highlights another level of plasticity of the cortex where the population of cINs in turn feedback on the IPs to control neurogenesis output.

Here, we propose that the cell-intrinsic regulation of pausing by CCP1 activity is an important driver of cINs migration, which acts in concert with attractive cues layered along migratory streams (Flames et al., 2004, Tiveron et al., 2006) to control the flow of cINs in the cortex. It would then be interesting to investigate whether diffusible cues attracting more migrating cINs modulate their pausing behavior. Together with previous work, our data suggest that a bidirectional molecular communication between IPs and cINs shapes neocortex histogenesis.

### Coordination of cINs Migration and Neurogenesis Might Be Relevant to the Diseased Human Brain

Pathogenic variants of *CCP1* (*AGTPBP1*) have recently been reported by the Decipher platform (Firth et al., 2009) for patients suffering from general delay of development including cognitive development (e.g., late acquisition of speech). More specifically, heterozygote deletions of parts of the *CCP1* locus (patients 269941 and 255872) as well as a heterozygote missense variant (chromosome 9, position 88207599 G → A) have been reported. It would be interesting to study human pathogenic *CCP1* variants in the context of cINs migration and PNs generation.

Increased brain volume and cortical thickness size have recently been described in patients suffering from autism spectrum disorder (ASD) (Courchesne et al., 2011, Zielinski et al., 2014) and may result from accumulation of PNs (Vaccarino et al., 2009). Because CCP1 cKO mice showed increased cortical number of Cux1^+^ PNs at P21, our new mouse line might by a relevant model to explore the cellular and molecular mechanisms of ASD or other neurodevelopmental disorder characterized by abnormal cortical neurons numbers.

## STAR★Methods

### Key Resources Table

**Table:** 

REAGENT or RESOURCE	SOURCE	IDENTIFIER
**Antibodies**		

Goat anti-GFP polyclonal	Abcam	#ab6673
Rabbit anti-CCP1 polyclonal	Proteintech	#14067-1-AP
Rabbit anti-GFP polyclonal	Torrey Pine Biolabs	#TP401
Rabbit anti-Somatostatin-14 polyclonal	Peninsula Laboratories	#T-4102
Mouse anti-Parvalbumin monoclonal	Swant	#PV235
Rabbit anti-active Caspase-3	Promega	#G7481
Rabbit anti-PhH3 polyclonal	Abcam	#ab5176
Rat anti-Ctip2 monoclonal	Abcam	#ab18465
Rabbit anti-Cux1 polyclonal	Millipore	#abe217
Goat anti-Sox2 polyclonal	Santa-Cruz	#sc17320
Rat anti-Tbr2 monoclonal	eBioscience	#14-4875-82
Rabbit anti-poly E polyclonal	Adipogen	#AG-25B-0030
Mouse anti-GT335	home made by Dr. Carsten Janke	N/A
Rabbit anti-Δ2-tubulin polyclonal	Merck	#Ab3203
Mouse anti-MLCK monoclonal	Sigma	#M7905
Rabbit anti-MLCK polyclonal	Gentaur	#MBS822161
Rabbit anti-MLC	Cell Signaling	#3672
Mouse anti-MLC phospho-Ser19 monoclonal	Cell Signaling	#3675
Rabbit anti-Ki67	Abcam	ab15580
Mouse anti-BrdU	Rockland Antibodies and Assays	#200-301-H50
Mouse anti-acetylated α-tubulin monoclonal	Sigma	#T6793
Rabbit anti-α-tubulin polyclonal	Abcam	#Ab18251
Mouse anti-β-actin peroxidase monoclonal	Sigma	#A3854
Sheep anti-mouse IgG HRP conjugate	GE Healthcare Life Sciences	#NA931
Sheep anti-rabbit IgG HRP conjugate	GE Healthcare Life Sciences	#NA934
Mouse anti-IgG	Sigma	#M4280

**Recombinant DNA**		

pCAGGS-LifeAct-Ruby	Roland Wedlich-Soeldne, DE	N/A
pCX-eGFP	X. Morin, FR	N/A
pCAGGS-PACT-mKO1	F. Matsuzaki, JP	N/A
pTRE-YFP MLCK-3E+	Carsten Janke, FR	N/A
pTRE-YFP MLCK-1E	Carsten Janke, FR	N/A
pCALSL-mir30-TTLL1	C.L. Cepko, US	N/A
pCALSL-mir30-Ctrl	C.L. Cepko, US	N/A
pCIG2-CRE-GFP	F.Guillemot; UK	N/A

**Chemicals, Peptides, and Recombinant Proteins**		

EGF	Preprotech	#AF-100-15
bFGF	Preprotech	#100-18
DNaseI	ThermoFisher Scientific	#D12372
Phalloidin 647	ThermoFisher Scientific	#A22287
CldU	Abcam	#ab213715
EdU Click-iT	LifeTechnologies	#C10338
annexin-V Cy5 conjugate	Abnova	#KA0712
N-cadherin-Fc chimera	R&D Systems	#6626-NC
Fibronectin	Sigma	#F4759
TruSeq Stranded Total RNA kit	Illumina	#RS-122-2201

**Critical Commercial Assays**		

RhoA activation ELISA	Cytsokeleton	#BK124-S
GFP ELISA (GFP-MultiTrap)	Chromotek	N/A
MLCK Kinase Enzyme System and ADP-Glo Kinase Assay	Promega	# V4497
Phospho-HER4/ErbB4 (panTyr) Sandwich ELISA	Cell Signaling	#13125
ML7 hydrochloride	Tocris	# 4310

**Deposited Data**		

Proteome	This paper	PRIDE: PXD006397
RNaseq	This paper	GEO: GSE104090

**Experimental Models: Cell Lines**		

HEK293T	ATCC	#CRL-3216
Neuro-2A	ATCC	#CCL-131

**Experimental Models: Organisms/Strains**		

Mouse CCP1 lox/lox	Carten Janke, FR	unpublished
Mouse TTLL1 lox/lox	Carten Janke, FR	unpublished
Mouse Nkx2.1 KO	Kusakabe et al., 2006	N/A
Mouse Dlx5.6 CRE	K .Campbell, US	N/A
Mouse R26R-EYFP	Jackson laboratory	Stock No:006148
Mouse Rosa 26/^CAGTdTomato^ (loxP-stop-loxP-Tomato)	Jackson laboratory	Stock No:007914

**Software and Algorithms**		

MATLAB	The MathWorks Inc	https://www.mathworks.com
ImageJ	NIH	https://imagej.nih.gov/ij/
ImageJ MTrackJ (ImageScience)	Erik Meijering	https://imagescience.org/meijering/software/mtrackj/
GraphPad Prism 6	GraphPad Software	https://www.graphpad.com

**Primers**		

Primers are listed in Table S1	IDT	N/A

### Contact for Reagent and Resource Sharing

Further information and requests for reagents may be directed to and will be fulfilled by Lead Contact, Laurent Nguyen (lnguyen@uliege.be).

### Experimental Model and Subject Details

#### Mouse genetics

All mice were handled according to the ethical guidelines of the Belgian Ministry of Agriculture in agreement with European community Laboratory Animal Care and Use Regulations (86/609/CEE, Journal Officiel des Communautées Européennes, L358, 18 December 1986) and with experimental protocol n°1393. Animal handling in the UK was carried out in accordance with the United Kingdom legislation (ASPA 1986).

For CCP1 conditional KO, exons 20-21 of *Agtbpb1* gene were flanked with loxP sites. Two month-old CCP1 conditional KO females were crossed with either Dlx5-6 Cre or Dlx5-6 Cre; Rosa-FloxStop YFP males of the same age. Pregnant females were sacrificed at the gestational day 13.5 and 14.5 and both male and female embryos were used for the analysis of the migration profile of WT or cKO cINs. E13.5 male and female embryos were also used in proteomic and genomic analysis. P0, P3, P5, P7, P8 and P21 mouse pups/adolescent were used to characterize the postnatal positioning of WT or cKO CCP1 as well as their intrinsic cell death profile and cortical positioning. Males and females were used between P0 until P8 and only males were used at P21. For TTL1 conditional KO, *Ttll1* gene was flanked with loxP sites and 2 month-old female mice were mated to NestinCRE male mice of the same age. Finally, 2 month-old *Nkx2.1* full KO male and female mice were used (Kusakabe et al., 2006). Pregnant females were sacrificed at the gestational day E13.5 and both males and female embryos were used in experiments involving this mouse model. Nkx2.1 Cre mice were purchased from Jackson’s Laboratories (stock number 008661) and backcrossed into MF1 genetic background. These mice were mated to Rosa-FloxStop TdTomato in order to visualize recombined cells. Two month-old Nkx2.1 Cre; Rosa-FloxStop TdTomato mice were mated to *Ccp1*^lox/lox^ mice of the same age in order to impair CCP1 function in MGE progenitors expressing *Nkx2-1* gene. Pregnant females were sacrificed at the gestational day E13.5 and both male and female embryos were used in the experiments involving this mouse line.

### Method Details

#### Generation of CCP1 cKO mice

The conditional mutant mouse line for *Ccp1* (on exons 20 and 21) was generated at the Mouse Clinical Institute (MCI, Illkirch, France). The targeting vector was constructed as follows. The 5′ (4.5 kb), 3′ (1.4 kb) and inter-loxP (1.9) fragments were PCR amplified and sequentially subcloned into an MCI proprietary vector containing the LoxP sites and a Neo cassette flanked by Flippase Recognition Target (FRT) sites (Figure S1A). The linearized construct was electroporated in 129S2/SvPas mouse embryonic stem (ES) cells. After selection, targeted clones were identified by PCR using external primers and further confirmed by Southern blot with 5′ and 3′ external probes. Two positive ES clones were injected into blastocysts, and derived male chimeras gave germline transmission. The excision of the neomycin-resistance cassette was performed *in vivo* by breeding the chimeras with a Flp deleter line (C57BL/6N genetic backgroundFLP under ACTB promoter). The Flp transgene was segregated by breeding the first germline mice with a WT C57BL/6N animal. For generation of CCP1 KO, *Ccp1-*floxed mice were crossed with transgenic mice expressing Cre recombinase under the control of a CMV promoter. Genomic DNA isolated from mouse tail snip was analyzed by PCR. Mice were genotyped by polymerase chain reaction (PCR) according to MCI protocols using GoTag polymerase (Promega) and 33 amplification cycles. The three primer pairs used are listed in the Table S1.

#### MGE explant culture on matrigel matrix

MGE explants were placed in MatTek Petri dishes (MatTek Corporation #P35G-0-20-C), previously coated with non-diluted Matrigel matrix (BD Biosciences #35427). Explants were covered with a thin layer of 1:2 Matrigel matrix diluted in neurobasal medium (NBM) (GIBCO #21103-049) supplemented with B27 containing vitamin A (1:50), penicillin/streptomycin (1:100) and L-glutamine (1:100). Explants were kept for 2h at 37 0C in a humidified incubator saturated with 5% CO_2_, before NBM was added.

#### MGE explant culture on feeder layer of cortical neurons

For some experiments, MGE explants were cultured on top of a feeder layer of cortical cells prepared one day before from homochronic WT cortices (2 embryos/dish). Cortical cells were mechanically dissociated using polished glass pipettes and plated in MatTek dishes previously coated with poly-ornithine (100μg/mL) and laminin (5μg/mL) according to manufacturer’s recommendations. Cortical cells were left adhere for 2h and cultured in 2mL of supplemented NBM.

#### Culture of cortical progenitors

MatTek Petri dishes were coated with poly-ornithine (100μg/mL) and laminin (5μg/mL) according to manufacturer’s recommendations. Cortices from E12.5 NMRI mouse embryos were dissected (around 14 cortices for 10mL of culture medium) and mechanically dissociated. The cell suspension was filtered using a strainer of 40μL and 800μL of cell suspension were added to the coverslip of MatTek dishes. After 2h, 1mL of DMEM-F12 medium supplemented with B27 containing vitamin A (1:50) penicillin/streptomycin (1:100), L-glutamine (1:100), EGF and bFGF (20ng/mL final). Cells were kept in a humidified incubator saturated with 5% CO_2_ for 24h. MGEs were microdissected and cut into 4 homogeneous pieces. Twenty pieces of MGE were plated in contact with cortical progenitors or on top of a Millicell insert and maintained in culture for a an additional period of 24h. After this period, 1.5 μL of 10mM EdU were added to each dish for a period of 30 min.

#### MGE explant electroporation

Embryos brains (E13.5) were exposed, by removing head tissue and skin, in a Petri dish containing ice-cold HBSS. After removal of the cortex, plasmids were pressure-injected in the MGEs using a FemtoJet injector (Eppendorf) and electroporation performed placing platinum P3 electrodes surrounding head tissue. Electroporation was performed using a BTX ECM830 electroporator. Electroporation conditions: 5 pulses, 50V, 50ms, 1 s interval. Electroporated MGEs were micro-dissected and cut into 4-6 small explant pieces.

#### Organotypic slice culture from embryos brains

Heads from E12.5 embryos were harvested in ice-cold HBSS containing Ca^2+^ and Mg2+ and embedded in 4% low-melting agarose (BIO-RAD #161-3112) in HBSS. 300 μm coronal brain slices were prepared using a LeicaVT1000S vibratome. Slices were cut inside a chamber containing ice-cold HBSS at a speed of 2mm/s and a using a blade cutting frequency of 4. Immediately after cutting, slices were placed on 0.4 μm millicel membranes inserted on MatTek dishes containing supplemented NBM. Slices were kept in culture for at least 24h before time-lapse recording and medium replaced before recording.

#### Time-lapse recordings

GFP-expressing interneurons were monitored for at least 5h under a Nikon A1 inverted confocal microscope. MatTek dishes containing organotypic brain slices or MGE explants were placed in a humidified chamber saturated with 5% CO2. Recordings were performed at a constant temperature of 37 oC and an average of 16-18 Z steps were obtained each 5 min. Explants electroporated with LifeAct-Ruby plasmid were imaged using resonant confocal mode every 15 s. For MLCK inhibition, ML7 hydrochloride was added to the culture medium at a final concentration of 10μM, 5h before the start of time-lapse recording.

#### Brain tissue collection and preparation

P0, P3, P5, P7, P8 and P21 mice were perfused intracardially with a solution of 4% PFA and dissected brains were post-fixed in 4% PFA overnight (ON).

Embryonic brains or heads were kept in 4% PFA ON, at 4°C after dissection. Brain tissue was dehydrated in phosphate buffer (PB) solution containing 20% sucrose ON at 4°C. Brains were embedded and included in PB containing 15% sucrose and 7.5% gelatin (for immunohistochemistry) or OCT (for *in situ* hybridization) and kept at −80°C until used. Embryonic brain slices were cut under a cryostat at 14 μm (for IHC) or 20 μm (for ISH) and slices from postnatal animals kept in an anti-freeze solution at −20°C.

#### Immunohistochemistry in brain slices

After gelatin or anti-freeze removal, brain slices were permeabilized in PBS containing 0.2% Triton X-100 and blocked in 10% of donkey serum (DS) (Jackson Immunoresearch). Primary antibodies were incubated ON and used at a concentration of 1:500 in a solution of PBS containing 0.2% Triton X-100 and 1% DS. EDU was revealed by Click-iT reaction following the manufacturer’s instructions.

#### Determination of Ts and Tc of Tbr2+ cells

Time-mated females were injected with CldU and EdU at E13.5. The time between injections was 1.5h. After a total time of 2h, females were sacrificed and embryo heads were fixed in 4% PFA. The brains were cut into series of 14 μm coronal slices. The tissue was pre-treated with 10 mM citrate buffer, pH 6.0 at 100°C for 20 min. After 3 washes in PBS, slices were permeabilized and blocked in PBS containing 0.2% Triton X-100 and 10% NDS. Primary antibodies were incubated ON at 4°C in PBS containing 0.2X Triton X-100 and 1% NDS: anti-Tbr2 1:250, anti-BrdU 1:250 and anti-Ki67 1:250. EdU was revealed using the Click-iT revelation kit.

#### Immunocytochemistry on MGE explants

Explants were fixed in 4% PFA for 20 min and cells permeabilized in a PBS solution containing 0.2% Triton X-100 and blocked with 10% DS for 1h at room temperature. Explants were incubated with primary antibodies overnight at 4°C, diluted in a PBS solution containing 0.2% Triton X-100 and 1% DS. G-actin was stained using fluorescent 10μg/mL DNaseI and F-actin was stained using 1:100 Alexa Fluor 647 phalloïdin.

#### Image Acquisition and analysis

Images were acquired using inverted confocal microscopes (Nikon A1 and ZEISS LSM 880 with Airyscan). All image analysis: fluorescence intensity measurements and cell counting were done using ImageJ software. Binning analysis and cell counting were done on at least 6 brain slices spanning from rostral to caudal cortical levels for each brain analyzed. Bin length was adapted for each slice to fit the thickness of the cortex (from ventral to pial surfaces) and a fixed width of 100μm was used at E13.5 and of 500μM for P21.

#### Western blot

Proteins from E13.5 GEs were extracted using Tris-HCl buffer (10mM Tris-HCl, pH 7.5, 150mM NaCl, 0.5mM EDTA, 0.5% NP-40) supplemented with protease, phosphatase and deacetylase inhibitors. Proteins were separated in 7% to 15% polyacrylamide gels. Migration of the proteins was performed in Tris-Glycin running buffer and transfer of proteins performed onto nitrocellulose membranes in a Tris-Glycin-methanol solution at 4°C. Membranes were blocked 1h in 5% non-fat milk or 3% BSA and then incubated with primary antibodies. The signal of secondary HRP antibodies was detected with ultrasensitive films after exposure of the membranes to ECL substrate Thermo Scientific #32106).

#### *In situ* hybridization

Slices were pre-treated in triethanolamine 100mM, pH 8.0 and acetylated by adding drop wise 0.25% acetic anhydride for 15 min. Slices were pre-hybridized with warmed hybridization buffer (Amresco #0973) for at least 60 min at 70°C. Probes were pre-denatured at 70°C and used at a concentration 1:100 in hybridization buffer and hybridization was performed at 65°C ON. Washes were made with pre-warmed washing buffer (50% v/v formamide 2% v/v SSC, 0.1% v/v Tween20). Probes used: *Ccp1* (Dr. Carsten Janke), *Nrg1* LG or CDR, and *Erbb4* (kind gift from Dr. O. Marin), *Gad67* (Dr. F. Guillemot), *Lhx6* and *Lhx7* (Dr. M. Grigoriou), *Dlx2* (Dr. J. Rubenstein); *Nkx2-1* (Dr. SL. Ang), *Mash1* (Dr. C. Goridis).

#### G/F actin fraction purification

4 GEs were lysed in a buffer containing 50mM PIPES, pH6.9, 50 mM NaCl, 5mM MgCl2, 5mM EDTA, 5% v/v glycerol, 0.1% NP-40, 0.1% Triton X-100, 0.1% Tween20, 0.1% β-mercaptoethanol, at RT and then incubated at 37°C. Cell debris and nuclei were removed by centrifugation at 2000 g, at RT. The supernatant was ultracentrifuged at 100000 g for 1h at 37°C in a Beckman TL-100 ultracentrifuge. Supernatant containing globular actin was removed and 10μM cytochalasin D in 450L water μL were added to the pellet containing the fibrilar actin fraction ON at 4°C. Equal amounts of each fraction were loaded in polyacrylamide gels and analyzed by western blot.

#### Fluorescent Activated Cell Sorting

E13.5 embryonic GEs/brains were collected or E15.5 cortices were dissociated with papain containing 50mM DNase I and filtered through a 40um filter before being subjected to analysis. GFP + cells were FACS-sorted using FACS ARIA III (BD Biosciences) with FACSDiva 8.0 software. Sorting was performed in purity mode with 85-nozzle size.

#### Shotgun proteome and LC-MS/MS analysis

For differential proteome analysis of ganglionic eminences obtained from WT and CCP1cKO mice, we used a proteomics shotgun approach in combination with post-metabolic peptide labeling using NHS esters of ^12^C_3_- or ^13^C_3_-propionate. In brief, four ganglionic eminences per condition were pooled and pulverized in liquid nitrogen, and the resulting tissue powders were suspended in lysis buffer containing CHAPS (50 mM sodium phosphate pH 7.5, 100 mM NaCl, 0.8% wt/vol CHAPS) with protease inhibitors added (Complete Protease Inhibitor Cocktail, Roche). Tissue suspensions from each condition were separately subjected to three rounds of freeze-thaw lysis and cleared by centrifugation at 16,000 g for 5 min at 4°C. Sample protein concentrations were determined for each condition using the Bio-Rad DC Protein Assay Kit (Biorad), and the amount of clear lysate containing 300 μg of proteins (approximately 0.5 mL of cleared lysate) were processed as follows. To denature proteins, guanidinium hydrochloride was added to each lysate to a final concentration of 4 M. Each sample was desalted in 20 mM triethylammonium bicarbonate pH 8.0 over a NAP-5 desalting column (GE Healthcare). Then, each protein mixture was digested overnight at 37°C with sequencing-grade endoproteinase LysC (endoLys-C, Promega) at an enzyme/substrate ratio of 1/400 (w/w). Afterward, the resulting peptides were labeled post-metabolically using isotopic variants of N-hydroxysuccinimide (NHS)-propionate (i.e., NHS ester of 12C3-propionate for the CCP1 cKO mice sample and 13C3-propionate for the WT mice). The propionylation reagents were quenched by the addition of 40 mM glycine and the peptide mixture heated for 60 min at 100°C to reverse possible O-propionylation of Ser, Thr and Tyr. Equal amounts of CCP1 WT and cKO peptide sample were mixed, and following oxidation of methionines, peptides were fractionated via RP-HPLC in 30 s intervals, as described previously. Following peptide fractionation, peptide fractions eluting 12 min apart were pooled to reduce the number of LC-MS/MS runs (24 per analysis).

The obtained peptide mixtures were introduced into an LC-MS/MS system, an Ultimate 3000 RSLC nano-LC (Thermo Fischer Scientific) in-line connected to a Q-exactive (Thermo Fisher Scientific), for MS analysis. Peptides were loaded on a trapping column (made in-house; 100 μm inner diameter x 20 mm, 5 μm beads, C18 Reprosil-HD, Dr. Maisch). After flushing from the trapping column, the sample was loaded on a reverse-phase column (made in-house; 75 μm inner diameter x 150 mm, 3 μm beads, C18 Reprosil-HD, Dr. Maisch) packed in the needle (PicoFrit SELF/P PicoTip emitter, PF360-75-15-N-5, New Objective). Peptides were loaded with solvent A (0.1% trifluoroacetic acid, 2% acetonitrile in water) and were separated with a linear gradient from 2% solvent A’ (0.1% formic acid in water) to 55% solvent B (0.1% formic acid, 80% acetonitrile in water) at a flow rate of 300 nL/min, followed by a wash reaching 100% solvent B for 15 min.

The mass spectrometer was operated in data-dependent, positive ionization mode, automatically switching between MS and MS/MS acquisition for the 10 most abundant peaks in a given MS spectrum. In the Q-exactive, full-scan MS spectra (m/z 375–1500) were acquired in the Orbitrap at a target value of 1E6 with maximum ion injection time of 80 ms, and a resolution of 60,000 at 200 m/z. The 10 most intense ions fulfilling a predefined criterion (AGC target 1E5 ions, maximum ion injection time of 60 ms, isolation window of 1.5 m/z, fixed first mass of 145 m/z, spectrum data type: centroid, underfill ratio 2%, intensity threshold 1.3E4, exclusion of unassigned, singly charged precursors, peptide match preferred, exclude isotopes on, dynamic exclusion time of 12 s) were subjected to tandem MS scans at a resolution 15,000 (at 200 m/z).

From the MS/MS data in each LC-MS/MS run, Mascot Generic Files were created using Mascot Distiller software (version 2.5.1, Matrix Science). In the generation of these peak lists, grouping of spectra was allowed in Mascot Distiller with a maximum intermediate retention time of 30 s, and a maximum intermediate scan count of 5 was used where possible. Grouping was done with a 0.005-Da precursor tolerance. A peak list was only generated when the MS/MS spectrum contained more than 10 peaks. There was no deisotoping, and the relative signal-to-noise limit was set at 2. These peak lists were then searched with the Mascot search engine (Matrix Science) using the Mascot Daemon interface (version 2.5.1, Matrix Science, http://www.matrixscience.com). Spectra were searched against the Swiss-Prot database with taxonomy set to *Mus musculus* (release 2015_05). Two different type of searches were performed: in one of them, the enzyme was set to semi-endoLys-C. In both cases, enzyme settings allowed for one missed cleavage, nonetheless allowing for cleavage when lysine was followed by proline. Variable modifications were set to pyroglutamate formation of N-terminal glutamine and acetylation of the N terminus. Methionine oxidation was set as a fixed modification. Determination of the light (^12^C_3_-propionyl) and heavy (^13^C_3_-propionyl) labeled peptides for further quantification was enabled using the quantitation option in Mascot. The mass tolerance on precursor ions was 10 ppm (with Mascot’s C13 option set to 1), and that on fragment ions was 20 mmu. The peptide charge was set at 2+ and 3+, and the instrument specification was set as ESI-QUAD. Only peptides that were ranked one, have a minimum amino acid length of eight and scored above the threshold score, set at 99% confidence, were withheld. The false discovery rate was calculated for every search and was always found to be lower than 1.5%.

#### Interneuron race in micro-fluidic devices (MFDs)

MFDs were copied from a mold following the chamber design as published in Gluska et al. (Gluska et al., 2016). Individual microfluidic devices were made using Sylgard 184 silicone elastomer kit (Dow Corning # 1060040_S1) mixing the two components in the proportion 1:15. A homogeneous mix of the silicone elastomer was obtained by subjecting the molds to vacuum (2h) and the polymerization was performed for 2h at 60°C. MFDs were adhered to glass coverslips. The glass was coated with 1mg/mL polylysine, 40μg/mL anti-mouse IgG antibody, 10μg/mL N-cadherin-Fc chimera and 10μg/mL fibronectin.

#### RNA Sequencing

Total RNA was extracted from GFP FACS-sorted cells from CCP1 WT and cKO GEs and purified on columns (Quiagen RNeasy mini kit). ARN quality was controlled with an Agilent 2100 Bioanalyzer. DNA libraries were prepared after depletion of ribosomal RNAs. Paired-end sequencing was done on NextSeq 500 Illumina. Sequences alignment was done on mouse genome using the Tuxedo workflow, using Bowtie2. RNaseq data can be found at GEO: GSE104090.

#### RNA extractions and qRT-PCR

Total RNA was extracted from transfected N2A cells. RNA extraction was performed using the RNA extraction kit (QIAGEN). Synthesis of cDNA was performed on total RNA with SuperScript III reverse transcriptase (Invitrogen) according to the manufacturer’s instructions. Resulting cDNA was used for quantitative PCR, using Faststart Universal SYBR Green Master (Roche). Thermal cycling was performed on an Applied Biosystem 7900HT Fast Real-Time PCR detection system (Applied Biosystems, Foster city, USA). The primers used are listed in Table S1.

#### Sandwich ELISA

10ug/ml of monoclonal MLCK antibody was adhered to 96 well PVC plates, ON at 4°C. Wells were washed and blocked with 2% milk in wash buffer (25mM Tris, 150mM NaCl, 0.05% Tween pH7.2). 125μg of total protein extracts were incubated with the MLCK antibody in the wells for 1h at RT. The plate was then used either to quantify the MLCK 1E and the 3E+ or to quantify the total amount of MLCK used in the test. Primary detection antibodies were incubated 1h with an anti-Δ2-tubulin antibody, with an anti-PolyE antibody or with an anti-MLCK antibody all used at a concentration of 5mg/ml. Detection of the signal was performed using secondary HRP antibodies (10ng/ml), incubated for 1h at RT. SuperSignal ELISA Femto Substrate (ThermoScientific #37075) was then applied to the wells and luminescence was measured using Luminoskan Ascent Microplate Luminometer (ThermoFisher Scientific). For quantification of RhoA activation levels, ELISA was performed using Cytsokeleton kit following manufacturer’s recommendations. Similarly, Phospho-HER4/ErbB4 (panTyr) Sandwich ELISA was performed following manufacturer’s recommendations.

#### MLCK Activity test

The YFP-MLCK 3E+ and 1E expressing plasmids, as well as GFP control plasmid, were transfected in HEK293T cells for overexpression. Protein extracts were then transferred in 96 wells ELISA plates coated with a monoclonal GFP antibody. After incubation and washes, MLCK activity was measured using ADP-Glo Kinase Assay Kit. The synthetic peptide MRCL3 is a MLCK specific substrate. The sequence of MRCL3 is derived from amino acid residues 11-24 of human myosin regulatory light chain MRCL3. Luminescence was measured using Luminoskan Ascent Microplate Luminometer (ThermoFisher Scientific). The amount of MLCK presents in each reaction was the determined using the sandwich ELISA protocol.

#### Plasmids

cINs from GEs explants were electroporated with the following plasmids: pCAGGS-LifeAct-Ruby (0.8μg/μl), pCALSL-mir30-TTLL1 and pCALSL-mir30-Ctrl (2μg/μl) the RNAi sequences used are TTLL1: GGACCUGAUGGUGAAGAAC; Ctrl: TACGCGCATAAGATTAGGG; pCX-eGFP; pCAGGS-PACT-mKO1(1μg/μl). HEK293T Cell line was transfected (TransIT-X2, Mirus) with 0.8μg of the following plasmids: pTRE-YFP MLCK-3E+ and pTRE-YFP MLCK-1E and pCX-eGFP. pTRE-YFP MLCK-1E encodes a truncated form of MLCK lacking the 7 C-term last glutamate residues whereas pTRE-YFP MLCK-3E+ encodes MLCK full length bearing 3 to 8 glutamates on its C-term. Neuro-2A cell line was transfected with 0.8μg/μL of pCALSL-mir30-TTLL1 or pCALSL-mir30-Ctrl and pCIG2-CRE-GFP. Interneurons were electroporated with 1μg/μL pEYFP-CCP1, 1μg/μL pEYFP-CCP1dead (Rogowski et al., 2010) or pCIG TdTomato (1μg/μL). Cortical progenitors were electroporated with (1 μg/μL) mbCherry provided by Dr. Xavier Morin.

### Quantification and Statistical Analysis

Statistical analysis was performed using Graph Pad Prism 6 or MATLAB software. Parametric or non-parametric t tests were used to compare statistical difference between two experimental groups. The choice between parametric and non-parametric t test was made after verifying normality of distributions on Graph Pad Prism 6. One way-ANOVA was used to compare the statistical difference between at least three experimental groups. Two-way ANOVA was used to compare the statistical difference between at least three experimental groups based on multiple characteristics (e.g., number of cells per cortical area within different genotypes). Several assumptions were made to use ANOVA: similar variance across experimental distributions, samples were obtained independently and within each sample the observations were sampled independently and randomly. The choice of the post hoc test following ANOVA was made according to the necessity of computing confidence interval for every comparison. Permutation test was used to simulate the displacement of surrogate cells to avoid repeating the experiments many times in order to test statistical significance. Results are expressed as mean ± SEM and the experimental n, the statistical test used and statistical significance are indicated in the figure legends. A significance level of 0.05 was used in the statistical tests. For every experiment the results were re-calculated independently by the main authors of this manuscript. More information concerning data quantification and analysis for different experiments can be found below.

#### RNA sequencing analysis

Gene expression analysis was performed using Cuffdiff program. Differences of gene expression in interneurons from WT or cKO CCP1 mouse embryos was calculated from log2fold change of fragments per kilobase of exon per million reads (FPKM) values. See Figures 1 and S1.

#### Proteome analysis

Identified peptides were quantified using Mascot Distiller Tool, version 2.4.3.3 (Matrix Science in the precursor mode. All data management was done in ms-lims and data integration was performed using R (http://www.R-project.org) embedded in KNIME. The results of analyses are shown as the peptide ratio of the light (L)-labeled (CCP1cKO mice) versus the heavy (H)-labeled sample (CCP1 WT mice). Robust statistics was applied to the base-2 logarithm values of the peptide ratios accepted as valid by Mascot Distiller. The median of the peptide ratio distributions of both experiments was corrected to zero. Further, peptide ratios being set as FALSE were verified by individual inspection. Protein ratios were then calculated by taking the median of the peptide ratios that identified that protein. To identify significantly altered proteins, robust statistics was applied, proteins falling within the 95% confidence interval and that were identified by at least two peptides were considered not to be affected by *Ccp1* conditional knock-out. The mass spectrometry proteomics data have been deposited to the ProteomeXchange Consortium (http://proteomecentral.proteomexchange.org) via the PRIDE partner repository with the dataset identifier PRIDE: PXD006397.

#### Kinematic simulations of migrating cINs / analysis of cINs migration in MFDs

Each surrogate cell moved in one direction following a movement profile of pauses and instantaneous speeds sampled randomly from their distributions in WT and cKO CCP1 cells. The proportion of pause to movement, averaged across surrogates, was kept identical to that observed in each group. Surrogates were simulated for 10 hours and at the end of the simulation time, the percentage of them having crossed 10 different displacement thresholds were calculated. The procedure was repeated for 20 realizations in each of which the pause onsets, durations and the instantaneous speeds for each surrogate was randomized. All simulations were done in MATLAB.

Since INs do not move in a straight line in corridors of MFDs, a least-squares line was fitted to each cell’s positions over time and its displacements along and perpendicular to this line were found. Next, maximum displacement threshold crossed by 75% of cells in each population was calculated at each time instant. At the end of recording time, statistical significance of the observed difference between values of this threshold for the CCP1 cKO group and for the CCP1 WT group was assessed by a permutation test. The null hypothesis was that the difference in the maximum displacement thresholds between the two groups is observed independently of the assignment of final displacement values to the cells of these two groups. Thus, the values of displacements, found at the end of recording, pooled from both groups were shuffled and assigned randomly to cells from each group to construct two surrogate sets of displacement values. The difference between maximum displacement thresholds crossed by 75% of the cells from each of the two surrogate sets was found and the procedure was repeated 10000 times to construct a distribution of surrogate values. By comparing where the observed difference falls relative to all the surrogate differences under the null hypothesis of an outcome independent of group assignment of displacement values, a p value was found. The calculation of p value for the difference in maximum displacement thresholds was done for 19 different values of percentage of cells. See Figure 5.

#### Time-lapse recording analysis

Parameters such as speed, directionality, nucleokinesis amplitude and frequency were analyzed using MTTrack plugin on ImageJ software. The area of actomyosin contraction was visualized with Fire look-up table and quantified using ImageJ software. Life duration of neuritic branches was manually assessed, by counting the number of frames during which a branch is visible. Maximum centrosome-nucleus distance was manually measured using ImageJ software between the PACT-KO signal and the center of the nucleus on the frame preceding the start of nucleokinesis. See Figures 1, 2, and S1.

##### Quantification of the TS and TC of Tbr2^+^ progenitors

Ts and Tc were calculated according to Ponti et al. (2013).

See Figure 6

##### Histological analysis

For all histological quantifications at least six rostro to caudal brain sections per brain from at least 3 independent animals were analyzed. The distance between slices was rigorously determined. Counting bins of 100μm, 200μm or 500μm were drawn in dorso to caudal regions of the selected slices. All cells were quantified within the bins and quantifications were averaged for all corresponding slices from same brain.

##### RT-qPCR analysis

The quantity of each mRNA transcript was measured and expressed relative to β-actin and Glyceraldehyde-3-Phosphate dehydrogenase (GAPDH). Results are expressed as percentage of control change.

##### Western blot analysis

The density of the bands revealed in photographic films was quantified using the gel quantification plugin on ImageJ software. Absolute values were normalized on loading control (tubulin, actin) bands of the same samples run in the same gel. Results are expressed as percentage of control change.

## Supplemental Information

Movie S1. Migration of E13.5 WT or cKO CCP1 cINs on Homochronic Mixed Cortical Feeder, Related to Figure 1 GFP-expressing cINs migrating away from MGE explants prepared from E13.5 CCP1 WT embryos. Duration of recording is 5h. GFP-expressing cINs migrating away from MGE explants prepared from E13.5 CCP1 cKO embryos. Duration of recording is 5h.

Movie S2. Migration of pLifeAct-Ruby Electroporated E13.5 WT or cKO CCP1 cINs on Homochronic Mixed Cortical Feeder, Related to Figure 2 pLifeAct-Ruby electroporated cINs undergoing nucleokinesis and prepared from E13.5 CCP1 WT embryos. Duration of recording is 1h. pLifeAct-Ruby electroporated cINs undergoing nucleokinesis and prepared from E13.5 CCP1 cKO embryos. Duration of recording is 1h.

Movie S3. Migration of pPACT-KO Electroporated E13.5 WT or cKO CCP1 cINs on Homochronic Mixed Cortical Feeder, Related to Figure 2 pPACT-KO electroporated cINs undergoing nucleokinesis and prepared from E13.5 CCP1 WT embryos. Duration of recording is 1h. pPACT-KO electroporated cINs undergoing nucleokinesis and prepared from E13.5 CCP1 cKO embryos. Duration of recording is 1h.

Movie S4. Migration of E13.5 cKO CCP1 cINs on Homochronic Mixed Cortical Feeder upon DMSO or ML7 Treatments, Related to Figure 2 GFP-expressing cINs migrating on cortical feeder cultures prepared from E13.5 CCP1 cKO MGE explants. Cultures were treated with DMSO for 5h prior to recording. Duration of recording is 5h. GFP-expressing cINs migrating on cortical feeder cultures prepared from E13.5 CCP1 cKO MGE explants. Cultures were treated with ML7 10 μM for 5h prior to recording. Duration of recording is 5h.

Movie S5. Displacement of Cell Surrogates with WT or CCP1 cKO Migration Parameters, Related to Figure 5 Displacement of all cell surrogates in both groups (Group A gray and Group B red); lighter marker shade indicates higher total displacement of a surrogate. Horizontal bars mark the highest displacement thresholds crossed by 75% of surrogates in each group. Duration of simulation is 600min.

Movie S6. Displacement of WT or CCP1 cKO cINs in Microfluidic Devices, Related to Figure 5 Aligned displacement of the CCP1 WT (gray) and CCP1 cKO (red) cINs populations during 120min of time-lapse recording. Horizontal bars mark the highest migration distance toward cortex crossed by 75% of all cells in each population.
