# Supplementary material for: Cell-Intrinsic Control of Interneuron Migration Drives Cortical Morphogenesis
Source: Cell. 2018 Feb 22;172(5):1063–1078.e19. doi: 10.1016/j.cell.2018.01.031 (PMC5847171; doi:10.1016/j.cell.2018.01.031)
Supplement: Document S1. Table S1 [file mmc7.pdf]

Supplementary table 1

List of primers used in this study.

Primer	Forward Sequence 5’-3’	Reverse Sequence 5’-3’
Ccp1 (1)	GTACACAGACTTTTAGGCTAATCTC	ACTTCCACATCTGCACTGTGGACAG
Ccp1 (2)	CATAAGAACCAGGTTCATTCTGTCC	TAGCATCATGTTAAAACTCCCTCCT
Ccp1 (3)	CATAAGAACCAGGTTCATTCTGTCC	ACTTCCACATCTGCACTGTGGACAG
Ttl1	GAA GTC CCT TGG CGG AGA AA	CAT GTA GGT GAC GGG GAC AA
Foxp2	AGTGTGCCCAATGTGGGAG	CATGATAGCCTGCCTTATGAGTG
Meis2	TTCTTGACTGGGCTTTCCCC	CAGCTCATCGTACCTTTGCG
Er81	GGACGGGATGCTTCAAGATT3	AGGCCATGAAAAGCCAAACT
Sall3	TGGAGGTATCTACGGACAAGG	GGGTCACATTGGTACTAGGCA
Zic2	GGGGAGAAACCTTTCCAGTG	CTCATGGACCTTCATGTGCT
Zic4	GACGTGAGCGAATGATGTTG	GGATCGGCCTTTCAGGATTT
Six3	CAAGAACAGGCTCCAGCATC	CTGGAGGTTACCGAGAGGAT
Lhx1	GAACCTAGGTGCCAAACGTA	AACCAGACCTGGATAACACG
